# Modulating Neutrophil Extracellular Trap Formation *In Vivo* with Locoregional Precision Using Differently Charged Self-Assembled Hydrogels

**DOI:** 10.1021/acscentsci.4c02198

**Published:** 2025-03-12

**Authors:** Tania L. Lopez-Silva, Caleb F. Anderson, Joel P. Schneider

**Affiliations:** Chemical Biology Laboratory, Center for Cancer Research, National Cancer Institute, National Institutes of Health, Frederick, Maryland 21702, United States

## Abstract

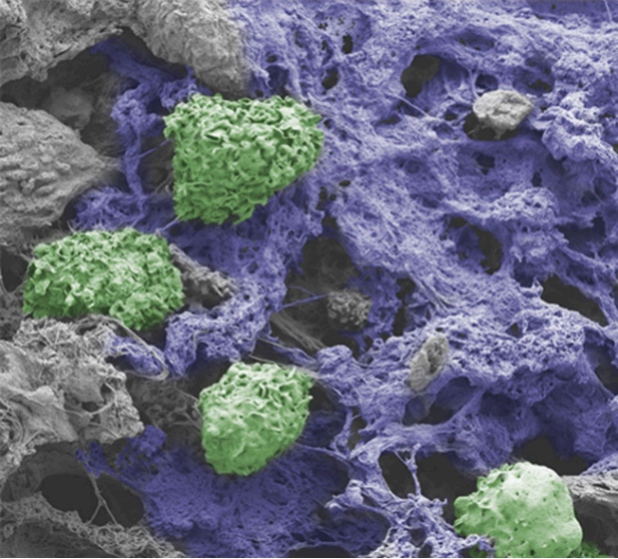

Neutrophil extracellular traps (NETs) are DNA networks released by neutrophils, first described as a defense response against pathogens but have since been associated with numerous inflammatory diseases. Diverse physical material properties have been shown to promote NET formation. Herein, we report the discovery that the charge of self-assembled peptide hydrogels predictably modulates the formation of NETs *in vivo* within the implanted material. Positively charged gels induce rapid NET release, whereas negatively charged gels do not. This differential immune response to our self-assembled peptide gels enabled the development of a material platform that allows rheostat-like modulation over the degree of NET formation with anatomical and locoregional control.

## Abstract 2

Self-assembled peptide-based hydrogel strategy for controlling the formation of neutrophil extracellular traps (NETs) *in vivo* with anatomical and locoregional precision, and the ability to regulate the degree of the response.

## Introduction

Biomaterials that modulate specific immune responses have shown potential for therapeutic and biomedical applications,^1−3^ including vaccines,^4−6^ cancer,^7,8^ wound healing,^9,10^ tissue regeneration,^11,12^ and immune tolerance.^13−15^ Commonly, materials facilitate the release of immunomodulating agents or induce an immune response by themselves based on their material properties and their interaction with immune cells upon implantation.^16^ The most well-known response to biomaterials is the foreign body reaction (FBR), in which the body induces inflammation and fibrosis to encapsulate the material. However, materials that induce alternate immune responses avoiding FBR are being developed by exploring how a material’s physicochemical properties influence the immune response.^14,17−21^ The rules governing material-immune responses are still being uncovered, and designing materials toward a targeted response remains challenging.

Neutrophils are the first responders to any injury or damage caused by the implantation of a material.^22^ The manner in which neutrophils interact with the material significantly influences the subsequent response ranging from tissue integration and repair to inflammation and FBR.^23,24^ Thus, controlling the neutrophil response to a material offers a potential avenue for directing the subsequent immunological reaction. One such response is the formation of neutrophil extracellular traps (NETs).^25,26^ NETs are fibrous networks composed of extracellular DNA and granule enzymes such as neutrophil elastase (NE) and myeloperoxidase (MPO), released upon neutrophil activation and death.^27^ Initially recognized as a defense mechanism against bacterial infection,^28^ NETs also play significant roles in inflammation,^29,30^ autoimmune diseases,^31,32^ cancer progression, and metastasis.^33−36^ Several factors can trigger NET formation, including bacteria, cytokines, danger-associated molecular patterns (DAMPs), lipopolysaccharides (LPS), phorbol myristate acetate (PMA), and monosodium urate crystals (MSU).^37−40^ In the context of sterile biomaterials, NET formation can be induced by the material’s properties, such as size and shape,^41^ diameter,^24^ hydrophobicity,^42^ roughness,^43^ and composition.^44^ While NET formation can be detrimental to healthy tissues and is linked to numerous pathologies, there is evidence that NETs can help resolve inflammation and protect against further tissue damage.^30^ Similarly, neutrophils and NETs have been shown to play a role in the efficacy of vaccines.^45−47^ Adjuvants, such as alum, rely on this response to induce immunity against antigens. Therefore, developing materials that can induce NETs in a controlled manner can potentially guide the immune response and holds the promise of studying NET formation in both disease and therapy with locoregional and temporal specificity.

**Figure 1:**
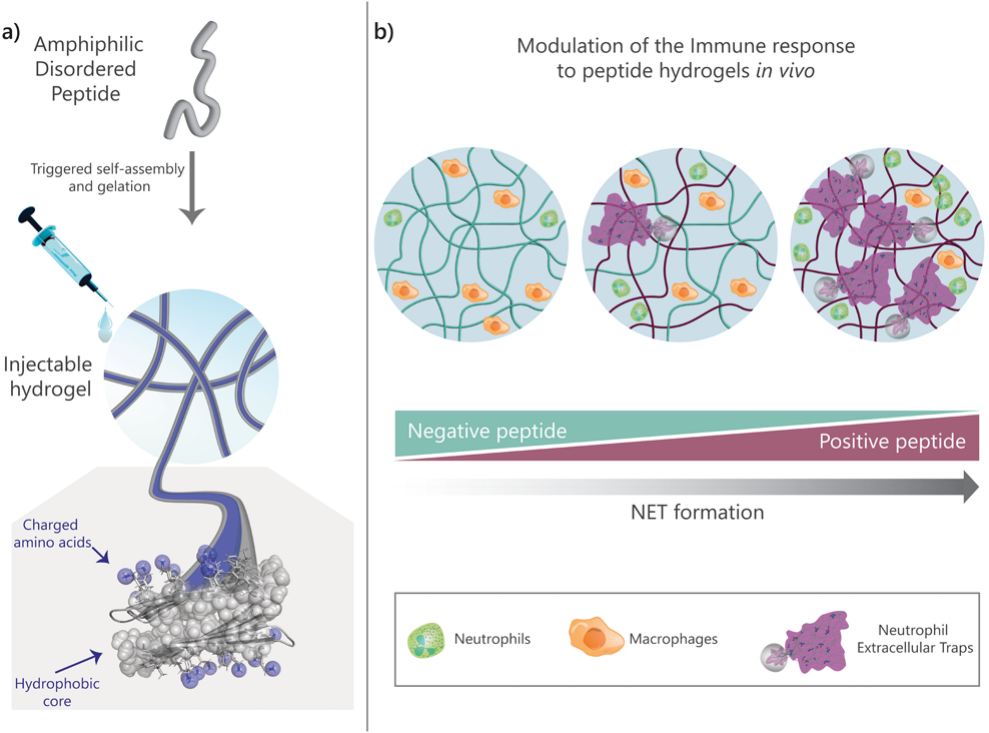
Modulating NET formation with self-assembling peptide hydrogels. (a) Hydrogels are composed of short amphiphilic peptides that self-assemble into β-sheet-rich nanofibrous networks. Implantation of peptide hydrogels by injection allows site-specific control of NET formation. (b) Highly positively charged peptide hydrogels induce NET formation directly within the hydrogel implant. In contrast, negatively charged gels have initial low neutrophil infiltration and do not induce NET release. The microscale precision and degree of NET formation can be tuned by creating gradients and composites of positive and negative peptide hydrogels.

We are currently studying how individual design features of peptide-based gels influence the immune response. During this investigation, we designed two oppositely charged gels to define the role of electrostatic potential and made the serendipitous discovery that highly positively charged gels quickly induce the formation of NETs *in vivo*. In contrast, gels carrying a negative charge do not elicit this same response. To our knowledge, the ability to induce NET formation using a gel’s electrostatic charge has not been reported and expands the list of material properties that can be exploited to induce this response. Based on this observation, we developed a peptide gel platform comprising easily injected materials that can be implanted into a targeted tissue to provide site-specific anatomical control over NET formation (Figure 1). Microscale locoregional control of NET release directly within a single implant is possible by modulation of the distribution of charge across the material. We also demonstrate that the degree of inflammation and NET formation *in vivo* can be controlled by employing composites of oppositely charged gels (Figure 1b). This study represents the first report of a peptide-material strategy to induce microscale NET formation *in vivo* with locoregional control and tunability at the tissue and implant level by using charge.

## Results and Discussion

### Designed Peptides Self-Assemble into Differently Charged Injectable Hydrogels

Our group has developed a family of amphiphilic peptides that self-assemble into nanofibers and form hydrogels when triggered by changes in solution pH, temperature, and ionic strength. Peptides assemble into bilayered cross β-sheet fibrillar structures in which each peptide adopts a facially amphiphilic β-hairpin conformation (Figure 1). A solid-state NMR structure of the fibrils formed by one of our previously designed peptides showed that the hydrophobic side chains of each peptide are packed into the dry core of the fibril and the hydrophilic side chains are solvent-exposed and available for interactions with the biological milieu (Figure 1).^48,49^ Herein, structure-based design was used to engineer peptides and their respective fibrils to direct the relative arrangement of charged residues within each peptide constituting the self-assembled fibrillar gel. This affords control over the presentation of charge and the gel’s electrostatic potential as a means to modulate the immune response. Our material design reduces biomaterial complexity, as the hydrogels are made of single peptides, which carry electrostatic potential that is amplified in the final self-assembled gel network.

**Figure 2:**
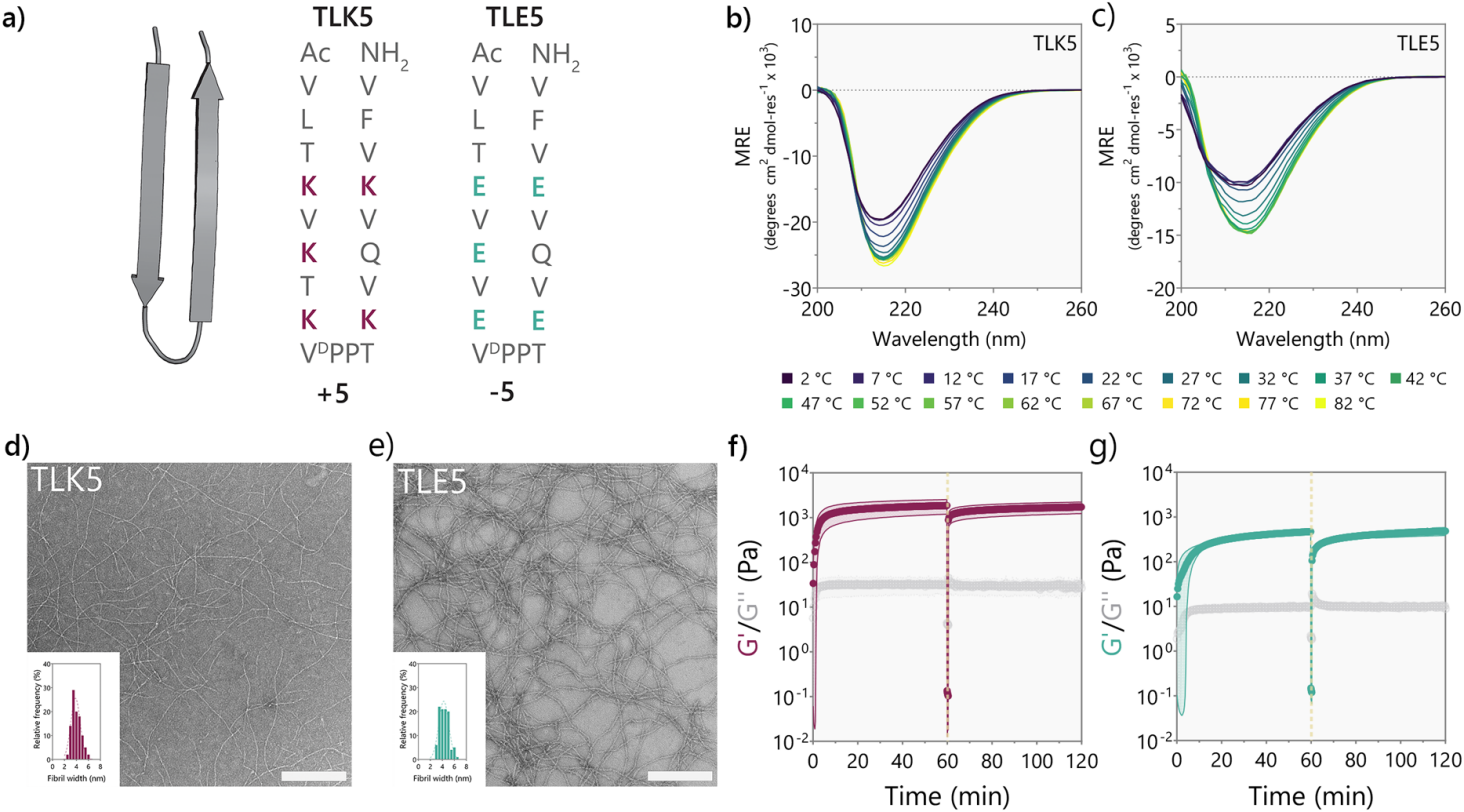
Structural and mechanical characterization of differently charged self-assembled peptide hydrogels. (a) Peptide sequences for TLK5 and TLE5 shown in β-hairpin conformations. (b, c) Circular dichroism spectroscopy spectra at 10 mg/mL (1 wt %) in 1× HBS at pH 7.4 at different temperatures for (b) TLK5 and (c) TLE5. (d, e) Negative-stain TEM images of (d) TLK5 and (e) TLE5 nanofibers and size distribution (inset). Scale bar represents 200 nm. (f, g) Viscoelastic properties of (f) TLK5 and (g) TLE5 1 wt % hydrogels in HBS at pH 7.4 over time. Hydrogels exhibit thixotropic behavior and can be injected easily as they recover after a shear event.

Figure 2a shows two β-hairpin peptides, named TLK5 and TLE5, that carry a primary charge of +5 and −5, respectively. Each peptide contains a four-residue reverse turn sequence (−V^D^PPT−) and a mixture of valine and threonine residues on their hydrophobic faces to facilitate peptide folding and hydrophobic effect-driven assembly. Importantly, each peptide also contains a block of five lysine or glutamic acid residues on their hydrophilic faces at sequence positions located near the reverse turn, where the conformation of the β-hairpins is likely to be less dynamic in their assembled state. A hydrophilic nonionic glutamine residue is also incorporated within each block. TLK5 and TLE5 peptides also contain a pair of leucine-phenylalanine residues at positions 2 and 19. These residues are known to form stabilizing pairwise interactions across β-sheets in naturally occurring proteins.^50^ Lastly, peptides are amidated at the C-termini and acetylated at the N-termini to avoid the presentation of charge at the peptide’s termini. These design features should result in peptides that undergo triggered assembly, forming gels whose fibrils display charge along their long axis (Figure 1a). All of the peptides in this study were prepared by Fmoc-based solid-phase peptide synthesis, purified by RP-HPLC, and characterized by LC-MS (Figure S1).

The potential of TLK5 and TLE5 to assemble, fold, and form fibrillar gel networks was assessed by a combination of spectroscopy, microscopy, and rheology. Both peptides readily form 1 wt % gels (10 mg/mL, 4.02 mM TLK5, 4.38 mM TLE5) in 1× HEPES buffer saline (HBS) at pH 7.4. Circular dichroism (CD) spectroscopy shows that both peptides adopt a β-sheet structure in the gelled state, presenting spectra with distinct minima at 216 nm (Figure 2b,c).^48^ Spectra show temperature-dependent behavior consistent with assembly and β-sheet formation being driven by the hydrophobic effect.^51,52^ Assembly is also concentration-dependent, with both peptides showing a low propensity toward β-sheet formation at lower peptide concentrations (μM range, Figures S2 and S3). TLK5 and TLE5 form long and flexible nanofibers with a similar morphology, as observed by TEM (Figure 2d,e). Fibrils have a diameter of around 4 nm, which is similar to the length of a folded β-hairpin and consistent with the structural model in Figure 1. Oscillatory rheology (Figure 2f,g) shows that hydrogel formation for both peptides is rapid at 37 °C. TLK5 forms a gel within seconds that further cures over about 14 min (Figure S4a), having a plateau storage modulus (*G*′) of 1860 ± 667 Pa and a loss modulus (*G*″) of 32 ± 13 Pa (Figure 2f, Figure S5a,b, and Table 1). TLE5 forms a more compliant gel with a *G*′ of 470 ± 63 Pa and a *G*″ of 10 ± 1 Pa that takes slightly longer to cure (Figure 2g, Figures S4b and S5c,d). Both peptides form viscoelastic materials (Table 1) that present thixotropic behavior (Figure 2f,g), flowing under high shear stress and recovering quickly after the cessation of shear. This property allows easy implantation by injection, facilitating anatomical control over gel placement. Overall, TLK5 and TLE5 form nanofibrous hydrogels with opposite formal charges that, as will be shown, can modulate NET formation in a charge-dependent manner *in vivo*.

**Table 1:** Viscoelastic Properties of Peptide Hydrogels

peptide/parameter	TLK5	TLE5
*G*′ (Pa)	1860 ± 667	470 ± 63
*G*″ (Pa)	32 ± 13	10 ± 1
tan δ	0.02 ± 0.001	0.02 ± 0.01
flow point (% strain)	52 ± 12	88 ± 61
recovery at 5 min (%)	71 ± 2	57 ± 7

### Differently Charged Gels Elicit Distinct Cellular Infiltration and Degradation Profiles *In Vivo*

**Figure 3:**
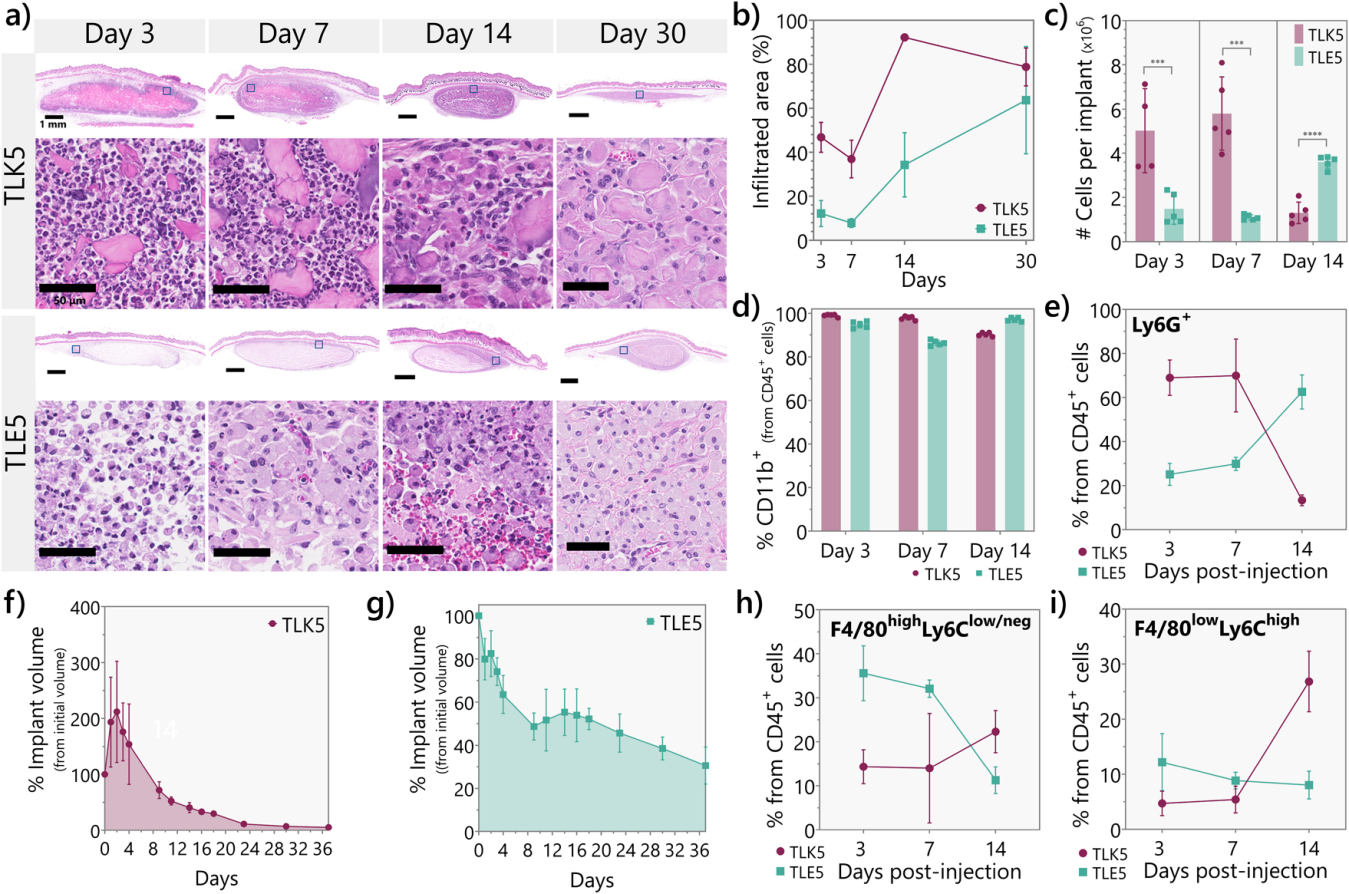
Immune response and degradation profiles of TLK5 and TLE5 hydrogels in a subcutaneous injection model. (a) H&E-stained tissue sections of implants at 3, 7, 14, and 30 days postinjection, demonstrating differential cellular infiltration patterns and evolution of the immune response. (b) Percentage of cell infiltrated area over time determined by histology, *n* = 3 mice. Statistical differences at day 3: TLE5 vs TLK5 **p-value < 0.003; at day 7: TLE5 vs TLK5 **p-value: 0.0037; at day 14: TLE5 vs TLK5 ***p-value: 0.0004. (c) Number of cells recovered per implant after digestion in preparation for flow cytometry. (d) Percentage of myeloid cells (CD45^+^CD11b^+^) in the different peptide implants. (e) Percentage of CD45^+^CD11b^+^Ly6G^+^ cells (neutrophils) from total leukocytes at different time points. Statistical comparison: day 3, TLK5 vs TLE5 *p*-value < 0.0001; day 7, TLK5 vs TLE5 *p*-value < 0.0005; day 14, TLK5 vs TLE5 *p*-value < 0.0001; *n* = 5 mice. (f, g) Degradation profile determined by ultrasound imaging for (f) TLK5 and (g) TLE5, *n* = 4 mice. (h) Percentage of CD45^+^CD11b^+^Ly6G^neg^F4/80^high^Ly6C^low/neg^ cells from total leukocytes at different time points. Statistical comparison: day 3, TLK5 vs TLE5 *p*-value < 0.0001; day 7, TLK5 vs TLE5 *p*-value: 0.0057; day 14, TLK5 vs TLE5 *p*-value < 0.0080; *n* = 5 mice. (i) Percentage of CD45^+^CD11b^+^Ly6G^neg^F4/80^low^Ly6C^high^ cells (monocytes/macrophages) from total CD45^+^ leukocytes for each peptide hydrogel at 3, 7, and 14 days postinjection. Statistical comparison: day 3, TLK5 vs TLE5 *p*-value: 0.0044; day 14, TLE5 vs TLK5 *p*-value < 0.0001; *n* = 5 mice. Error bars represent standard deviation.

We first investigated if, indeed, material charge could influence the immune response toward TLK5 and TLE5 gels using a murine subcutaneous injection model. This model is commonly used to evaluate material biocompatibility and immune response *in vivo* because it facilitates fast screening of different materials.^53−55^ Further, for injectable gels that are minimally invasive, it provides an uninjured tissue environment, minimizing additional inflammation and tissue damage.^20^ Hydrogels were harvested at 3, 7, 14, and 30 days postinjection and evaluated by histology. By day 3, the positively charged TLK5 gel was infiltrated by a large number of polymorphonuclear cells, mostly at the material’s periphery (Figure 3a–c), observed as a purple ring in the H&E-stained tissue section. The peptide gel is stained pink due to its eosinophilic character provided by the lysine residues. In the infiltrated area, cells are migrating toward the core and surrounding gel fragments. By day 7, the TLK5 implants show areas of dense cellular infiltration, but the core of the gel remains unpopulated. By days 14 and 30, the implant is fully infiltrated, and the gel is being degraded, phagocytosed, and remodeled by macrophages with a characteristic foamy phenotype. Collagen is deposited within the hydrogel implant, and its loose structure is similar to that of native subcutaneous tissue (Figure S6). More than 90% of the infiltrating cells are of myeloid origin (Figure 3d, Figure S7), with around 70% being neutrophils (CD45^+^CD11b^+^Ly6G^+^ cells) at days 3 and 7 (Figure 3e), but by day 14, there is an increase in the percentage of monocyte/macrophage populations (CD45^+^CD11b^+^F4/80^high/low^ cells) as shown in Figure 3h,i. Two distinct populations of monocyte/macrophages are observed in TLK5 gels (Ly6C^high^ and Ly6C^low/neg^), suggesting the infiltration of macrophages with diverse phenotype, including pro-inflammatory, pro-repair, and tissue resident macrophages.^56−59^ By day 14 there is an increase of F4/80^int/low^Ly6C^high^ cells, which have been reported to have phagocytic activity (Figure 3i).^60,61^

In contrast, the negatively charged TLE5 gel had significantly fewer infiltrating cells that are located primarily at the periphery (Figure 3a–c) during the first week after implantation. The majority of these cells are macrophages with characteristic large nuclei and cytoplasm and an F4/80 positive phenotype (Figure 3h,i). Only a few polymorphonuclear cells are observed (Figure 3e). At later time points (days 14 and 30), the TLE5 implant presents more cellular infiltration with dense areas at the periphery of the implant surrounding a significantly less infiltrated core. Collagen is being deposited in the highly infiltrated areas (Figure S6). Interestingly, 2 weeks after injection, the implant contains a higher percentage of neutrophils (Figure 3e). This is unexpected, as neutrophils are known to be early responders that attract monocytes and macrophages. However, for the TLE5 gel, we see that although the surface contains more macrophage-like cells (Figure 3a, Figure S8), at later time points the core of the material is filled with red blood cells, neutrophils, and other immune cells. The high neutrophil content at day 14 postinjection could be due to accumulation of neutrophils from the blood present in the core of the TLE5 implants.

The presence of distinct cell types over time suggests that the degradation rates of the oppositely charged gels should differ. As such, we measured the differences in the degradation times for the TLK5 and TLE5 gels using ultrasound imaging (Figure S9). This technique allowed us to follow the implant volume over the course of 38 days in the same animal with a single injection. TLK5 implants showed an increase in volume in the early days postinjection, with a peak at day 2 of around 200% from the initial volume (Figure 3f). This increase in volume is due to acute inflammation caused by the peptide hydrogel, characterized by high cellular infiltration, as seen by histology, and irritation with blood vessels surrounding the material, as observed by gross histology (Figure S10). After the initial inflammation, the hydrogel volume decreased over time, with 7.3 ± 3.4% of the initial volume remaining after a month. At 37 days postimplantation, almost all the peptide hydrogel is fully degraded and replaced with normal subcutaneous tissue with loose collagen, nerve fascicles, blood vessels, and adipose tissue (Figure S6). In contrast, TLE5 implants have a steady and much slower degradation rate compared with that of the TLK5 gel (Figure 3g). The TLE5 gel did not present the initial increase in volume, in agreement with the histological data showing minimal inflammation (Figure 3a, Figure S10). In fact, 30.6 ± 8.6% of the TLE5 implant volume remains after 37 days, emphasizing the difference in degradation profiles between the two peptide gels carrying opposite charge. Overall, the TLK5 and TLE5 gels elicit divergent immune responses with different cellular infiltrates and degradation profiles, demonstrating the impact of material charge on the host immune response.

Material stiffness is known to also affect the immune response to biomaterials.^23,62^ There is a 4-fold difference in the *G*′ of TLK5 vs TLE5 gels. Control studies using two previously developed electropositive peptide gels, HLT2 and MAX8 (Figure S11), show that charge, as opposed to stiffness, is driving the differential immune response. Both control gels are significantly softer than the TLK5 gel yet carry similar charge. Further, the *G*′ value of the MAX8 gel is similar to that of the negatively charged TLE5 gel, providing a second control. The MAX8 peptide has a formal charge of +7 and forms a gel with a *G*′ of around 500 Pa,^63^ whereas HLT2 (+5) forms a more compliant gel with a *G*′ of 233 Pa.^64^ Both MAX8 and HLT2 gels elicit an inflammatory response similar to that of the TLK5 gel (Figure S12) and degrade within the same time frame.^63,65^ Further, divergent responses are observed between the MAX8 and TLE5 gels, which have similar *G*′ values. Thus, material charge is most likely driving the immune response to the materials studied herein.

### Neutrophils Release Neutrophil Extracellular Traps (NETs) within Positively Charged Gel Implants

**Figure 4:**
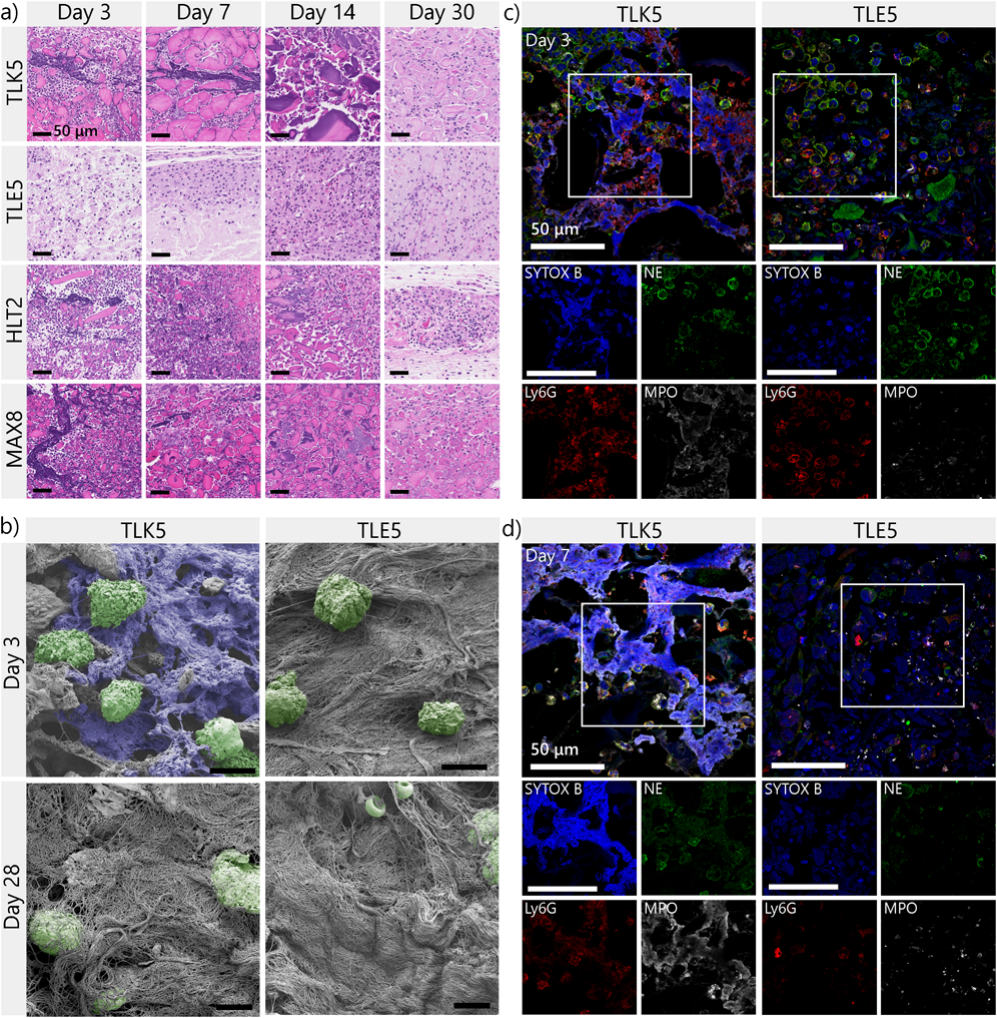
NETs formed within the implants of positively charged gels. (a) H&E staining of implant slides at different times. The positive peptide gels have fibrous-like purple structures, indicating extracellular DNA within the implants. These DNA networks are not observed in TLE5. (b) SEM images of implants to observe the NETs and implant morphology 3 and 28 days postinjection. Cells in the field of view are colored green; amorphous network structures that do not correspond to collagen or typical ECM/gel morphology are colored blue. Scale bar is 6 μm. (c, d) Representative immunofluorescence images stained for NETs markers NE (green), MPO (white), DNA (blue), and Ly6G (red) at days (b) 3 and (c) 7 postinjection. Quantitative assessment of enzyme levels cannot be determined from these images, as they are derived from localized regions of an implant. Quantification of enzyme levels in whole implants by ELISA is included in Figure 5. Scale bar is 50 μm.

Close inspection of the histology data at days 3 and 7 showed the unexpected formation of distinct fibrous networks at the periphery of the positively charged gels (TLK5, HLT2, and MAX8; Figure 4a, Figures S13–S15). These networks are formed around small gel fragments that are separated by small channels that may facilitate neutrophil infiltration. Hematoxylin staining showed that the web-like structures were basophilic, consistent with the presence of extracellular DNA, and the temporal formation of these networks correlated well with the early appearance of high numbers of neutrophils at the implant, suggesting that they could be NETs (Figure 3e). Correlation of the histology, flow cytometry, and subsequent immunofluorescent staining of implant sections strongly supports the assertion that these web-like structures are indeed NETs (Figure 4c,d, Figures S16–S19). At day 3, microscale networks of extracellular DNA (blue) colocalized with the granulocytic enzymes, myeloperoxidase (MPO, white), and to a lesser extent neutrophil elastase (NE, green). These enzymes are present in implant areas having a high neutrophil density and neutrophil debris (Ly6G, red). These hallmarks of NET formation are even more pronounced at day 7, especially in the TLK5 gel (Figure 4d, Figure S19). In stark contrast, no NETs were observed in the negatively charged TLE5 gel at any time point, indicating that the material charge is likely the differentiating factor (Figure 4a, Figure S8). TLE5 implants display only normal intracellular DNA staining within the nuclei of infiltrating cells, with a subset of these cells staining positive for intracellular NE, MPO, and membrane-bound Ly6G (Figure 4c,d, Figures S16–S19).

The differential immune response to the oppositely charged gels (TLK5 vs TLE5) was further examined by scanning electron microscopy (SEM). Implants were analyzed at their periphery, where NETs were prominently observed by histology. At day 3, web-like networks are clearly observed in the TLK5 implant (Figure 4b, Figure S20). These structures are absent in native gels that have never been implanted (Figure S20b). The peptide gel itself appears as a featureless gray mat by SEM in both native and implanted materials. For TLK5 implants, we also observe small channels surrounding gel fragments filled with amorphous web-like structures, consistent with NET formation (Figure S20a). SEM also highlights the presence of infiltrating cells proximal to the observed filamentous networks. In contrast, these amorphous fibrous structures were absent in the TLE5 implant, which presented fewer cells that were surrounded by more structured collagen bundles. At day 28, amorphous networks are no longer observed in the TLK5 implant, which now contains collagen and cells. These latter observations coupled with the histology data suggest the resolution of NETs en route to gel degradation and tissue remodeling. Taken together, the histology, flow cytometry, and SEM data suggest that the positively charged gels induce the recruitment and swarming of neutrophils upon material implantation, which interact with the material and release NETs. This early time event most likely passivates material charge and defines later immune events, leading to the resolution of inflammation.

**Figure 5:**
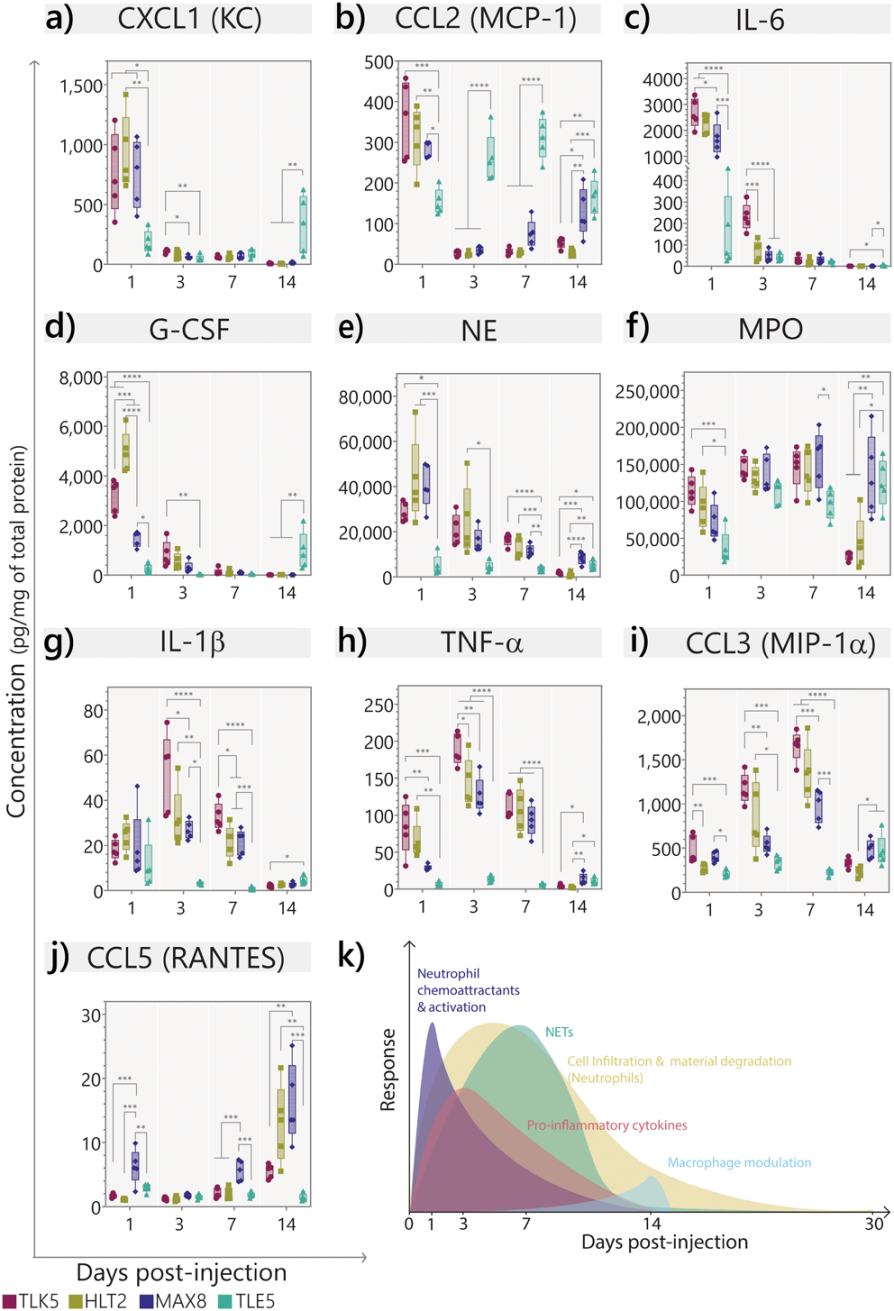
Evolution of signaling molecules involved in neutrophil recruitment and NET formation. (a–j) Concentration of enzymes, cytokines, and chemokines implicated in neutrophil recruitment, activation, and NET formation at 1, 3, 7, and 14 days postinjection. Data are represented as a range with minimum and maximum values, and the mean is represented as a line. *n* = 5. Statistical comparisons are shown in the Supporting Information. (k) Representation of the immune response elicited by TLK5.

Next, we analyzed the biomolecular milieu within the implants to understand how material charge influences the production of cytokines and enzymes involved in neutrophil recruitment, activation, NET release, and resolution of the response. In this experiment, implants were harvested over the time course of the immune response, and proteins were obtained from the implant lysates. Relevant signaling molecules required for neutrophil migration and NET formation are present at high levels in the positively charged gels shortly after implantation. CXCL1, a potent neutrophil chemoattractant secreted by tissue resident macrophages,^66−68^ is significantly higher in all three positive gels in comparison with the TLE5 gel at day 1 but rapidly decreases by day 3 (Figure 5a). This CXCL1 burst facilitates rapid recruitment of neutrophils immediately after gel injection, which then infiltrate the periphery of the implant, as shown by histology. A similar trend is observed for the monocyte chemoattractant CCL2 (MCP-1), the pro-inflammatory cytokine IL-6, and the granulocyte colony-stimulating factor (G-CSF), with elevated levels at day 1, followed by a decrease as the acute inflammatory response evolves (Figure 5b–d). Importantly, G-CSF has been shown to activate and prime neutrophils for NET formation, suggesting that neutrophils are activated to release NETs as early as day 1.^69,70^ Further, increased levels of NE and MPO, granule enzymes that regulate the formation of NETs, are observed at day 1 (Figure 5e,f).^28,71,72^ These enzymes are commonly bound to the DNA comprising NETs, which we observed by histology from day 3 through day 7. In the case of MPO, initial concentrations among groups show less pronounced differences (Figure 5f). However, TLK5 and HLT2 gels exhibit higher MPO levels at day 1 postinjection. By days 3 and 7, MPO levels are similar across all materials, which may be attributed to the presence of macrophages and other myeloid cells that produce MPO in all the gels.^73,74^

Levels of the pro-inflammatory cytokines IL-1β and TNF-α are moderate in the positively charged gels at day 1 but increase and peak at day 3 (Figure 5g,h). We also observe high concentrations of the macrophage inflammatory protein-1α (MIP-1α) that peak at day 7 (Figure 5i). Neutrophils produce this chemokine as a chemotactic for monocytes and neutrophils, and it is relevant for the progression of the inflammatory response elicited by the positive gels.^75^ In fact, we see an increase in the monocyte/macrophage percentage at day 14 for these materials, possibly due to the release of MIP-1α by neutrophils. All of these cytokine levels decrease with the concomitant increase in CCL5 at day 14. CCL5 is involved in lymphocytes and macrophage reprogramming to the resolution phase of inflammation (Figure 5j).^76^ Taken together, this suggests that the positively charged materials induce an acute inflammatory response that resolves over time.

In contrast, the negatively charged TLE5 gel exhibited low and constant levels of CXCL1 and G-CSF through the first week postinjection. However, by day 14, the concentration of both chemokines increased, which correlates with the higher percentage of infiltrating neutrophils observed at the core of the implant at that time point (Figure 3). Levels of CCL2 in TLE5 lysates progressively increase and peak around days 3–7, presenting significantly higher levels in comparison to the cationic gels. CCL2 regulates monocyte and macrophage recruitment, and the high levels of this chemokine strongly correlate with the higher percentage of macrophages and monocytes present in the implant at days 3 and 7, as seen by flow cytometry. Interestingly, TLE5 lysates contain moderate levels of MPO that could originate from the neutrophil and monocyte/macrophage populations infiltrating the implant.^77^ MPO levels increase by day 14 as more neutrophils infiltrate the core of the material. These results suggest that TLE5 induces a latent neutrophil response, where the high neutrophil and cytokine content could be the result of the slow degradation rate of the gel and of cells and blood accumulation at the core of the gel implant, as seen by histology (Figure S8). Importantly, the levels of cytokines and markers associated with inflammation (i.e., TNF-α, IL-1β, IL-6, NE) remain low in TLE5 over the course of the study, and the infiltrating neutrophils do not produce NETs (Figure S21) even at days 14 and 30 postinjection. Thus, there is a divergent immune response to the TLE5 gel in comparison with the other positively charged gels.

Figure 5k summarizes the temporal immune response to the positively charged gels, correlating all the data collected thus far. These gels elicit an acute inflammatory response immediately after injection that is characterized by a fast recruitment and activation of neutrophils, the formation of NETs observed at days 3 and 7, and the recruitment of monocytes and macrophages as the response progresses. Importantly, we observe the resolution of inflammation accompanied by gel degradation and elimination of NETs within 1 month (Figure 5k). Thus, for these positively charged gels, NET formation is not followed by chronic inflammation, tissue damage, or the FBR. The differential immune response observed as a function of material charge opens the possibility of developing a material platform capable of controlling the location and degree of NET formation.

### Locoregional Control of NET Formation

**Figure 6:**
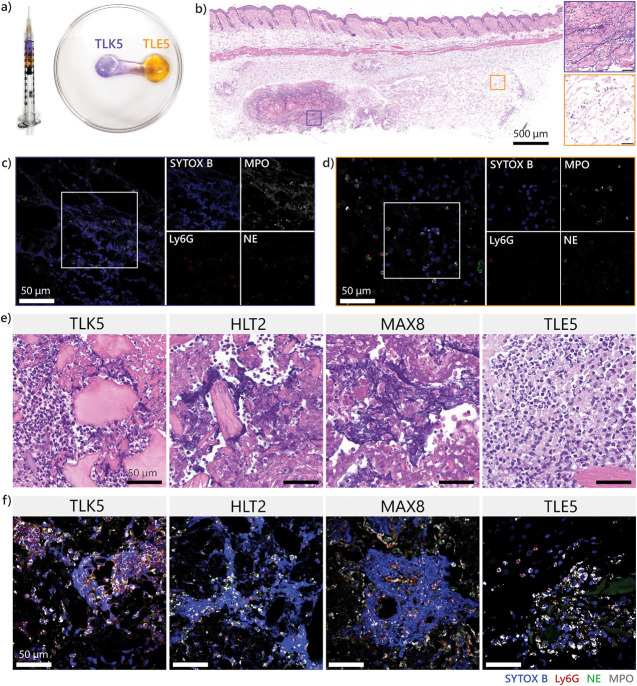
Locoregional control of NET formation. (a) Charge distribution within a single implant is controlled by sequential loading and injecting TLK5 and TLE5 gels via a syringe. (b) H&E-stained tissue section of the resulting implant containing two domains of oppositely charged gels 3 days postinjection. The inset outlined in purple shows a region of the TLK5 domain. The orange inset shows a region enriched with the TLE5 gel. (c) Immunofluorescent staining for DNA, Ly6G, NE, and MPO shows that NETs formed in the TLK5 domain. (d) Immunofluorescent staining of the TLE5 domain. (e) H&E-stained tissue sections of peptide gels injected in the gastrocnemius muscle showing the presence or absence of NETs 3 days postinjection. (f) Merged immunofluorescent images of intramuscularly injected gels stained for NET markers.

The contrasting immune responses triggered by the oppositely charged TLK5 and TLE5 gels allow precise control of NET formation within a single implant. The distribution of charge can be controlled by taking advantage of the rheological properties of these gels. Separate TLK5 and TLE5 gels can be sequentially drawn into a syringe and then delivered to a common site with minimal mixing (Figure 6a). This results in the delivery of a single implant with two separate domains: one containing TLK5 enriched with positive charge and the other containing negative charge provided by TLE5. Once implanted, distinct immune responses are elicited by the spatially distinct TLK5 and TLE5 domains (Figure 6b). One side of the implant has high cellular infiltration by polymorphonuclear cells and contains NETs, similar to the immune response observed in implants of TLK5 alone (Figure 6b,c). Conversely, there is lower cell infiltration and the absence of NETs in the TLE5-containing domain (Figure 6b–d). Thus, inflammation and NET formation can be controlled with locoregional specificity by simply engineering the delivery of two oppositely charged materials.

Anatomical control of NET formation is possible by the facile delivery of the gels into a tissue of interest by a simple injection. We performed intramuscular injections of all the gels into the mouse gastrocnemius muscle and assessed NET formation by histology at day 3 (Figure S22). All of the materials were highly infiltrated by cells, but only the positively charged gels contained areas with NETs, as confirmed by H&E (Figure 6e) and immunofluorescent staining (Figure 6f). NETs are observed only within the implant and do not form in the muscle fibers, demonstrating microscale locoregional control over the response (Figure S23). However, the muscle around the gel implant appears to have more cellular infiltration than the control muscle without injection (Figure S23). Although histology suggests that the immune responses elicited in the muscle and the subcutaneous space are likely not identical,^55^ neutrophil recruitment and NET release are conserved in both anatomical sites early in the immune response. It would certainly be interesting to assess if this is the case in other tissue types, which is an area of future work.

### Controlling the Degree of NET Formation by Modulating Material Charge

**Figure 7:**
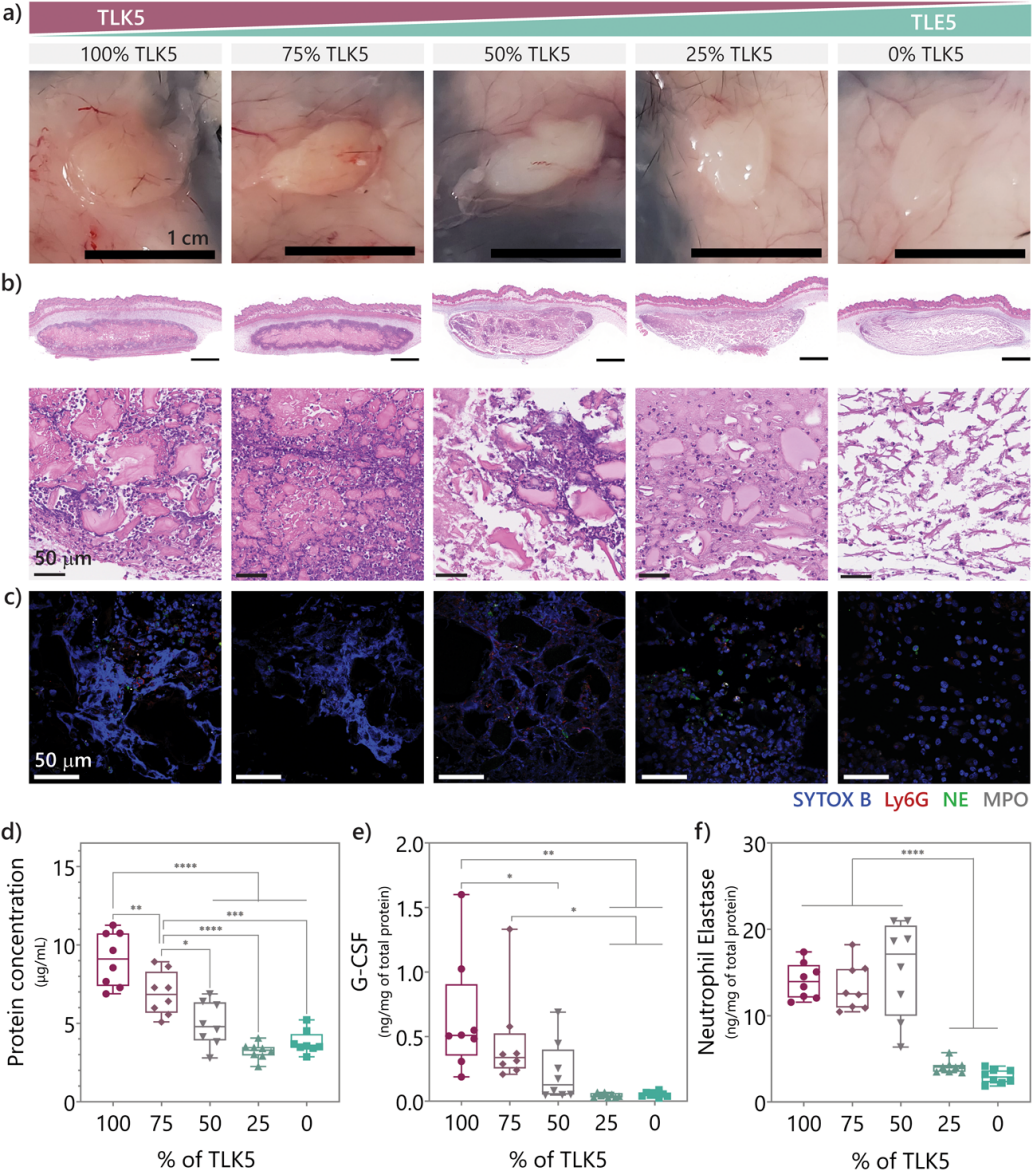
Composites gels of varying charge enable fine-tuning of the degree of inflammation and NET release. (a) Gross histology of implants of TLK5-TLE5 composites ranging from 100% TLK5 to 0% TLK5 3 days postinjection. (b) H&E-stained tissue sections of the different composites implanted subcutaneously. (c) Immunofluorescent-stained images of the different implants for DNA, Ly6G, NE, and MPO. (d) Protein concentration of lysates from the different composites. Data are represented as a range with minimum and maximum values, and the mean is represented as a line. *n* = 8. Statistical comparisons: *p-value: 0.0301; **p-value: 0.0096; ***p-value: 0.0001; ****p-value < 0.0001. (e) Concentration of neutrophil elastase present in the implant lysates with different TLK5 content. Data are represented as a range with minimum and maximum values, and the mean is represented as a line. *n* = 8. Statistical comparisons: ****p-value < 0.0001. (f) Concentration of G-CSF in the implant lysates with a range of TLK5 percentages. Data are represented as a range with minimum and maximum values, and the mean is represented as a line. *n* = 8. Statistical comparisons: 100% vs 50% *p-value: 0.0383; 100% vs 25% **p-value: 0.0013; 100% vs 0% *p*-value: 0.0015; 75% vs 25% *p*-value: 0.0416; 75% vs 0% *p*-value: 0.0484.

Given that material charge is a key determinant of the neutrophil response, we hypothesized that the degree of inflammation and NET formation could be tuned by adjusting the overall charge displayed within the gel network. To test this hypothesis, we prepared composite materials where charge was systematically varied by first preparing separate TLK5 and TLE5 gels and then combining them at different volume ratios (100, 75, 50, 25, and 0% TLK5 relative to TLE5) by shear-thin mixing. This preparation method results in gel composites with a range of positive charge content. Mixing oppositely charged gels results in slight turbidity proportional to the TLK5 content (Figure S24), indicating possible nanofiber aggregation due to electrostatic interactions. However, the injectability of the resulting materials is not affected. Each gel composite was injected subcutaneously, and the immune response was evaluated at day 3 when NETs were first observed for the TLK5 gel. Notably, histology showed that fine control over the degree of inflammation could be achieved by modulating the material charge (Figure 7a). Gross histology images show a spectrum of implant appearances that range from the characteristic irritation and yellow coloration, previously observed for the 100% TLK5 gel, to a clear implant as seen before for the 100% TLE5 gel. We also observe a rheostat-like variation on the degree of cellular infiltration by H&E staining (Figure 7b). The composite with 75% TLK5, which is predominantly positively charged, shows an infiltration profile similar to that of pure TLK5, where polymorphonuclear cells infiltrate into the periphery of the implant using channels that surround gel fragments. In contrast, the composite with 25% TLK5 has lower cell infiltration and resembles the pure TLE5 implants more. The composite comprising an equal mixture (50%) of TLK5 and TLE5 has a distinct appearance, with areas of high cellular infiltration mixed with areas with low cell density (Figure 7b). This suggests that the implant displays charge heterogeneously, having distinct microscale domains of positive and negative charge. This heterogeneity is probably a reflection of the mixing efficiency of the two gels. We observed similar trends with respect to NET formation. NETs are observed in the 100, 75, and 50% TLK5 composites by immunofluorescent staining (Figure 7c). NETs are formed at the periphery of the 75% TLK5 composite, similar to the pure TLK5 gel. Interestingly, for the 50% TLK5 composite, NET formation appears to take place only in the microscale domains that contain high cell density (Figure 7b). Here, DNA networks are confined into distinct regions that are surrounded by domains with fewer cells and no NETs. Lastly, no NETs were observed in the 0% and 25% TLK5 composite gels.

This rheostat behavior is also observed at the level of secreted protein and NET biomarkers measured from implant lysates isolated on day 3. Total protein, G-CSF, and NE, which are involved in neutrophil activation and NET regulation, vary according to the amount of TLK5 gel present in each composite. Total protein levels, an indicator of cell infiltration, decrease in nearly a stepwise fashion as the amount of TLK5 in each composite is decreased (Figure 7d). With respect to neutrophil activation, composites containing higher amounts of TLK5 gel (50–100%) have higher levels of G-CSF (Figure 7e). Of note, in this experiment, G-CSF levels are measured at day 3. Higher levels most likely exist at earlier times, as was observed for the pure TLK5 gel on day 1 (Figure 5d). This suggests that priming of neutrophils to release NETs may take place early for the composite gels as well. Composites with higher TLK5 content (50–100%) also had higher levels of NE in comparison to the 0–25% TLK5 composites (Figure 7f). As expected, NETs are observed by histology in composite implants having higher concentrations of NE (Figure 7b,c,f). These data demonstrate that formulating composite gels of varying positive charge affords control over the degree of inflammation and NET formation.

## Conclusion

Herein, we found that peptide gel charge determines neutrophil behavior and NET formation *in vivo*. The highly positively charged gel formed by the self-assembling peptide TLK5 induces an acute inflammatory response defined by the recruitment and infiltration of neutrophils that then release NETs within the implanted material. Over time, inflammation resolves, and the gel and NETs are cleared by infiltrating macrophages. In contrast, the negatively charged gel formed by peptide TLE5 elicits low cellular infiltration and no NET formation. Highlighting the impact of gel charge, we show that this material property allows for spatiotemporal control over NET formation in subcutaneous and intramuscular tissues. NET formation can even be controllably induced within specific regions of a single implant via the differential presentation of opposite charge. Further, the degree of inflammation and NET formation can be controlled by employing composite gels of varying positive charge. Thus, peptide gel charge affords control over NET release *in vivo* and offers the promise of studying NET formation in both disease and therapy with locoregional specificity.

## Experimental Section

### Peptide Synthesis and Purification

MAX8, HLT2, TLK5, and TLE5 were synthesized using a CEM Liberty Blue microwave-assisted peptide synthesizer on ProTide Rink amide resin (0.58–0.65 mmol/g) at a 0.25 mmol scale. Fmoc deprotection cycles were performed with 20% piperidine in DMF (15 s at 75 °C followed by 50 s at 90 °C). The first 11 couplings (residues 20-10) were performed at 90 °C for 4 min using Fmoc-protected amino acids (5 equiv, 0.2 M in DMF), Oxyma (5 equiv, 1 M in DMF), and DIC (5 equiv, 0.5 M in DMF). The valine after the d-proline amino acid was triple coupled with HCTU (5 equiv, 0.45 M in DMF) and DIPEA (10 equiv, 2 M in NMP) for 30 min at room temperature. The same coupling conditions were used for the subsequent two residues with double coupling. The remaining six residues were coupled at 90 °C as described above. MAX8 and HLT2 have free N-termini. For TLK5 and TLE5, the N-terminus was acetylated with acetic anhydride (10% v/v in DMF). Resin was washed with DCM and DMF and dried under vacuum before cleavage. Peptides were cleaved with 25 mL of 95% trifluoroacetic acid (TFA), 2.5% triisopropylsilane, and 2.5% water for 3 h. TFA was removed by argon gas flushing, and crude peptides were precipitated with cold diethyl ether, followed by two washes. Crude peptides were dissolved in water and lyophilized before mass confirmation and purification.

Peptides MAX8, HLT2, and TLK5 were purified by preparative RP-HPLC on a Waters 600 system equipped with a Waters 2489 or 2487 UV/vis detector using a Vydac 218TP C18 column (250 × 4.6 mm, 5 μm) at a flow rate of 8.0 mL/min and a column temperature of 40 °C. The UV trace was monitored at 220 nm. Gradients and solvent systems for preparative RP-HPLC were as follows: MAX8:100% Standard A (0.1% TFA in H_2_O) for 2 min, followed by a linear gradient from 0 to 21% Standard B (90% acetonitrile, 9.9% H_2_O, 0.1% TFA) in 5 min (4.2% Standard B/min). Then, a linear gradient of 0.5% Standard B/min was used for 40 min. MAX8 eluted at 36% Standard B. HLT2:100% Standard A for 2 min, followed by a linear gradient from 0 to 19% Standard B in 5 min (3.8% Standard B/min). Then, a linear gradient of 0.5% Standard B/min for 40 min. HLT2 eluted at 34% Standard B. TLK5:100% Standard A for 2 min, followed by a linear gradient from 0 to 23% Standard B in 5 min (4.6% Standard B/min). Then, a linear gradient of 0.5% Standard B/min for 40 min. TLK5 eluted at 38% Standard B. Peptide purity was assessed by LC-MS (Shimadzu LCMS 2020) using a Phenomenex Luna C18 column 100 Å (150 × 3 mm, 5 μm) with a linear gradient from 0 to 90% Standard B2 (0.1% formic acid in acetonitrile) at 2% B2/min and a column temperature of 40 °C. Purity was also assessed by analytical RP-HPLC (Agilent 1200 series) using a Vydac 218TP C18 column (250 × 4.6 mm, 5 μm) at 40 °C with a linear gradient from 0 to 100% Standard B at a rate of 1% B/min. Peptides were lyophilized to afford a white powder. TLE5 was purified by preparative RP-HPLC using a Water 600 system with a 2489 UV/vis detector in a Phenomenex PolymerX RP-1 column (250 × 21.2 mm, 10 μm), heated to 40 °C at 8 mL/min. The gradient was 100% Standard C (20 mM NH_4_HCO_3_) for 2 min, followed by a linear gradient from 0 to 16% Standard D (80% acetonitrile, 20% 20 mM NH_4_HCO_3_) in 5 min (3.2% Standard D/min). Then, a linear gradient of 0.5% Standard D/min was used for 40 min. TLE5 eluted at 31% Standard D. Purity was assessed by LC-MS and analytical HPLC as previously described but with a Phenomenex PolymerX RP-1 column (250 × 4.6 mm, 10 μm) using gradients of 1% Standard D/min. After lyophilization, TLE5 was converted to sodium salt by dissolving the peptide in water and adjusting the pH to 7.4 with the addition of aqueous NaOH. The peptide solution was frozen and lyophilized again to afford a white powder. All peptides were dissolved in ultrapure or endotoxin-free water and sterile-filtered with a 0.2 μm polystyrene filter before being lyophilized again for their use in further experiments.

### Peptide Characterization

Peptides MAX8 and HLT2 have been characterized and published previously.^78,79^ TLK5 and TLE5 are new designs and were fully characterized for their secondary structure, self-assembly, gelation, and viscoelastic properties, as described below.

### Circular Dichroism (CD) Spectroscopy

Characterization of the secondary structure and β-sheet formation as a function of buffer and temperature was performed by using a Jasco J-1500 CD spectrometer. Ice-cold peptide stock solutions in ultrapure water at 300 μM or 2 wt % (8.03 mM TLK5, 8.76 mM TLE5) were mixed with ice-cold 2× HEPES buffer solution (HBS, 50 mM HEPES, 300 mM NaCl, pH 7.4) for a final concentration of 150 μM or 1 wt % (4.02 mM TLK5, 4.38 mM TLE5). The samples were analyzed in a 1 mm (for 150 μM) or 0.1 mm (for 1 wt %) high precision cuvette (Hellma Analytics). The samples were allowed to equilibrate on the spectrometer for 10 min at 2 °C. Then, wavelength scans from 260 to 200 nm were taken every 5 °C. Between every temperature interval, the samples were allowed to equilibrate for 10 min before the measurement. Data were converted to mean residue ellipticity (MRE), calculated as
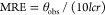
where θ_obs_ is the ellipticity in millidegrees, *l* is the light path on the cuvette, *c* is the molar concentration (M), and *r* is the number of peptide bonds (residues) in the peptide.

### Transmission Electron Microscopy (TEM)

First, 1 wt % peptide gels were prepared by mixing 2 wt % peptide solutions in ultrapure water with 2× HBS (1:1). Samples were cured overnight at 37 °C. An aliquot of peptide gel was diluted 40-fold for TLK5 and 100-fold for TLE5 in ultrapure water and mixed thoroughly. Then, 3.5 μL of the diluted gel was deposited on top of a glow discharge-treated grid (400-square mesh, CF400-CU, Electron Microscopy Science, Inc., PELCO easiGlow, Ted Pella, Inc.) and allowed to sit for 1 min. The grid was blotted with clean filter paper and washed three times with ultrapure water by adding a 3.5 μL water droplet on top, followed by blotting with paper. Samples were negatively stained with 3.5 μL of 2 wt % uranyl acetate in water. Samples were dried overnight at room temperature before imaging was performed on an FEI Tecnai 12 Twin transmission electron microscope at 80 kV acceleration voltage. Images were captured with an AMT XR16 charge-coupled device (CDC) bottom-mount camera (Advanced Microscopy Techniques, Corp.). The fibril width was measured using ImageJ (NIH).

### Oscillatory Rheology

Characterization of gelation and viscoelastic properties of the peptide gels was performed using a Discovery HR20 instrument (TA Instruments) with a 25 mm stainless steel parallel plate geometry and 500 μm gap height. Peptides were dissolved in ice-cold ultrapure water (20 mg/mL) and diluted 1:1 with ice-cold 2× HBS immediately before the test. Then, 300 μL of 1 wt % (10 mg/mL) peptide solution was immediately placed on the cold rheometer stage at 5 °C. Geometry was lowered to the measurement gap and covered with mineral oil (ASTM oil standard) to prevent drying. The sample was allowed to soak at 5 °C for 10 s, followed by a temperature ramp to 37 °C (17.45 °C/min) at a strain of 0.2% and angular frequency of 6.0 rad/s. The evolution of the storage (*G*′) and loss (*G*″) moduli was monitored for 1 h with an oscillatory time sweep experiment at 37 °C, 0.2% strain, and a 6.0 rad/s angular frequency. Then, 1000% strain was applied for 30 s, followed by another oscillation time sweep at 0.2% to evaluate the thixotropic properties and recovery of the peptide gels. To evaluate the time dependence of the viscoelastic properties, oscillation frequency sweeps were performed on the same sample at 37 °C, 0.2% strain, and a frequency range from 0.1 to 100 rad/s. Then, oscillation amplitude seeps were performed at 37 °C, a 6.0 rad/s angular frequency, and a strain range from 0.1 to 1000% to determine the viscoelastic behavior and flow point of each gel. To estimate the *G*′ stabilization time, the first derivative of *G*′ as a function of time was calculated using GraphPad Prism. The stabilization time was determined at the time where there is a ≤10% change in the highest first derivative value.

### Peptide Gelation for *In Vivo* Experiments

After purification, peptides were dissolved in endotoxin-free water, sterile-filtered through a 0.22 μm PVDF Durapore Membrane (Millex-GV Merck Millipore, Tullagreen, Ireland), and filtered through a Mustang E 0.2 μm, 25 mm Acrodisc unit under aseptic conditions. Peptides were then lyophilized and stored at −20 °C until use. Sterile-filtered peptide powders were dissolved in cold ultrapure water under sterile conditions at 2 wt % (20 mg/mL). After complete peptide dissolution, prechilled sterile and endotoxin-free 2× HBS (50 mM HEPES, 300 mM NaCl, pH 7.4) was added to the peptide solution in a 1:1 ratio. The final concentration of the hydrogel was 1 wt % (10 mg/mL) in 25 mM HEPES, 150 mM NaCl, pH 7.4. Peptide solutions were loaded into sterile syringes, placed in sterile pouches, and transferred to an incubator at 37 °C overnight to promote complete gelation. Endotoxin levels were quantified for each gel using the ToxinSensor Chromogenic LAL Endotoxin Assay, Endotoxin Detection System (GenScript). No endotoxins were detected for HLT2 and MAX8, and TLK5 and TLE5 had levels below or equal to 0.1 EU/mL, which is the limit of detection for this protocol. Osmolarity was measured using a Vapor Pressure Osmometer (VAPRO, Wescor). The osmolarity was within a range of 353–387 mmol/kg.

### *In Vivo* Subcutaneous and Intramuscular Injection Model

All experimental procedures were approved by the NCI Animal Care and Use Committee (ACUC) and performed according to the Animal Welfare Act and NIH guidelines for the care and use of laboratory animals. Female C57BL/6J mice (8 to 12 weeks old) were obtained from The Jackson Laboratories. Animals were anesthetized in an induction chamber using 3–4% isoflurane (1–1.5 L/min oxygen flow rate). Then, animals were placed in a surgery board at 37 °C with eye lubricant, and anesthesia was maintained at 2% isoflurane. Hair from the back or leg was removed using clippers, followed by the application of a facial hair removal cream. The cream was removed, and the area was neutralized with 1% (v/v) acetic acid solution to prevent irritation. The mouse skin was cleaned with alcohol swabs before injection. Hydrogels were injected into the subcutaneous space of their dorsal flank using a 27-gauge needle or into the gastrocnemius muscle, and the animals were allowed to recover in a heating pad with food, water, and/or DietGel recovery. At specific time points during the study, animals were anesthetized with 4–5% isoflurane and euthanized by CO_2_ asphyxiation, followed by a secondary method of euthanasia before tissue harvesting.

For histological analysis, mice received two 150 μL injections to the left and right sides of their dorsal flanks. For intramuscular injections, mice received a 50 μL injection in the right gastrocnemius muscle. Animals were euthanized at 3, 7, 14, and 30 days postinjection. For flow cytometry and cytokine/chemokine quantification, mice received four 100 μL injections. For NET modulation studies, mice received four 100 μL injections, and implants from the same mouse were used for both histological analysis and cytokine/chemokine studies. For degradation studies, mice received one 150 μL injection in the right flank. For locoregional control studies, TLE5 peptide gel was drawn first into the syringe to a final volume of 50 μL. Then, TLK5 was drawn next carefully to prevent bubbles to a final volume of 50 μL, resulting in a single syringe with two different materials, as shown in Figure 6a. Hydrogels were injected slowly into the subcutaneous space to prevent the mixing of both materials *in vivo*.

### Degradation Studies Using Ultrasound Imaging

First, 150 μL of peptide gel was subcutaneously administered on the lower back of 6–8 week old C57BL/6J mice (*n* = 4). Hydrogel implant degradation was evaluated by B-mode ultrasound imaging using a Vevo2100 preclinical scanner (VisualSonics Inc., Toronto, Canada) at 1, 2, 3, 4, 5, 10, 12, 15, 17, 19, 24, 31, and 38 days postinjection. Ultrasound images were acquired with an MS 550S (40 MHz) linear array transducer with a step size of 0.076 mm. Hydrogel volumes were measured for each time point using the parallel contour algorithm (Vevo LAB software, ver. 1.7.1, VisualSonics Inc., Toronto, Canada). Imaging was performed by the Small Animal Imaging Program at the NCI Frederick.

### Histological Analysis and Immunofluorescence Staining

After tissue harvesting, implanted hydrogels on the skin were fixed with 10% neutral buffered formalin overnight. The skin and implants were trimmed, cut, and sent for processing, sectioning, and staining to Histoserv, Inc. (Germantown, MD, USA). Hematoxylin and eosin- and Masson’s trichome-stained slides were imaged using an Aperio Slide Scan (40×) and deposited to Indica Laboratories HALO Link by the Molecular Histopathology Laboratory at the NCI Frederick.

5 μm tissue slides were deparaffinized with two xylene treatments for 5 min each, followed by rehydration with a series EtOH treatments (from 100% to 50% EtOH in DI water). Antigens were retrieved by boiling in sodium citrate buffer (10 mM sodium citrate, 0.05% Tween 20 at pH 6.0). Then, slides were rinsed and permeabilized twice with 0.1% PBST (0.1% Tween 20 in 1× PBS at pH 7.4) for 2 min and blocked with 1% w/v BSA in PBS for 30 min. Tissue sections were incubated with primary antibodies (Table S1) overnight at 4 °C and then rinsed with PBST twice and incubated with secondary antibodies (Table S1) for 1 h at room temperature. Tissue slides were rinsed and incubated with SYTOX Blue for 30 min before a last wash session and mounting with ProLong Diamond Antifade reagent (Invitrogen). Slides were analyzed using a Zeiss LSM780 Laser scanning confocal microscope at the Optical Microscopy and Analysis Laboratory at the NCI Frederick.

### Flow Cytometry and Immunophenotyping

Hydrogel implants were harvested after 3, 7, and 14 days postinjection (four implants per animal) with any connective and fat tissue trimmed. Then, implants were cut with a scalpel and digested with 5 mL of Liberase (Thermolysin Medium, 0.2 WU/mL) and 80 U/mL DNase in 1× PBS at 37 °C for 45 min with gentle agitation. After digestion, the cell suspension was filtered through a 40 μm cell filter. The filter was rinsed several times with 1% BSA 2 mM EDTA in DPBS to a final volume of 20 mL. Cell suspensions were centrifuged for 5 min at 1200 rpm at 4 °C, washed with 1× PBS once, centrifuged, and resuspended in 1× PBS. Cells were counted, concentration adjusted, and 1 × 10^6^ cells were placed in each tube for staining (Table S2). Cells were incubated with 100 μL of fixable live/dead Zombie NIR (1:2000 dilution) in PBS for 20 min on ice, followed by two washes with stained buffer (FBS in DPBS, BD Biosciences). Then, cells were blocked with Mouse BD Fc block CD16/CD32 for 5 min at 4 °C before the antibody cocktail was added (Table S2) to a final volume of 100 μL. Cells were incubated on ice for 30 min, washed twice with stain buffer, and fixed with BD Cytofix fixation buffer (200 μL for 15 min on ice) while being filtered through a 30 μm cell trainer, followed by a last wash. Cells were resuspended in stain buffer and analyzed using a Cytek Aurora Spectral Flow Cytometer, and data were analyzed on SpectroFlo. Gates were determined using fluorescent minus one (FMO) controls.

### Scanning Electron Microscopy of Implants and Native Gels

SEM sample preparation and imaging were performed by the Center for Cancer Research Volume Electron Microscopy (CvEM) core at the NCI. Peptide implants were harvested from the mice as described previously at days 3 and 28 postinjection. Implants were trimmed and cleaned, followed by fixation with a solution of 4% formaldehyde and 2% glutaraldehyde (v/v) in 0.1 M cacodylate buffer, pH 7.4. Implants were subsequently stained and fixed using a 1% osmium tetroxide solution and broken to reveal the periphery area of the implants, where NETs are typically observed by histology. Then, samples were dehydrated using a standard alcohol dehydration process ranging from 35% to 100% with final dehydration completed using tetramethylsilane. The dried samples were coated with an iridium layer of approximately 5 nm using an Emitech K575X sputter coater. Samples were imaged with a ZEISS GeminiSEM 450 instrument. The same sample preparation procedure was performed in TLK5 and TLE5 native hydrogels to observe the morphology of the materials before injection and *in vivo* modifications.

### Multiplex Immunoassay for Cytokine and Chemokine Quantification

Peptide hydrogels (1 wt %, 10 mg/mL) were prepared under sterile conditions as described before. Mice received four 100 μL peptide gel injections at the dorsal aspect (*n* = 5 mice per time point). At 1, 3, 7, and 14 days postinjection, mice were euthanized according to the ACUC approved protocol. Hydrogel implants (four implants) were retrieved from the subcutaneous space, trimmed, rinsed with cold PBS, and placed on 1 mL of 1× Halt protease inhibitor cocktail (Thermo Scientific) in ice-cold PBS. Implants were cut into small pieces and homogenized before Triton X-100 (IBI Scientific) was added and homogenized again. Proteins were extracted from the samples by freeze–thaw lysis by fast freezing samples in liquid nitrogen, followed by thawing on ice and pulsed vortexing. Lysates were centrifuged at 10 000*g* for 5 min at 4 °C, and supernatants were aliquoted for storage at −80 °C for protein quantification. Protein concentration as determined with the Pierce BCA Protein Assay Kit (Thermo Scientific) in 1:10 diluted samples with a working range of 20–2000 μg/mL, as specified by the manufactured protocol.

Cytokines and chemokines were quantified using a custom mouse panel LEGENDplex bead-based multiplex immunoassay kit for CXCL1 (KC), CCL3 (MIP-1α), CCL5 (RANTES), IL-1β, IFN-γ, IL-6, G-CSF, TNF-α, IFN-β, CCL2 (MCP-1), and GM-CSF. Samples were prepared according to the manufacturer’s instructions. Briefly, lysates were diluted 1:2 with an assay buffer prior to quantification. All samples and reagents were warmed to room temperature and run in duplicate. 25 μL of assay buffer and sample or standard was added to a V-bottom plate with 25 μL of mixed beads. Samples were incubated for 2 h at room temperature under constant shaking, followed by a wash step before incubation with biotinylated detection antibodies for 1 h at room temperature under shaking. Then, 25 μL of SA-PE was added to each sample without washing and incubated for 30 min at room temperature under constant shaking. Samples were washed, transferred to 5 mL Corning Falcon round-bottom polystyrene tubes, and analyzed in a Cytek Aurora spectral flow cytometer. Data were analyzed using Biolegend’s LEGENDplex data analysis software.

ELISA assays were used to quantify neutrophil elastase, myeloperoxidase, and G-CSF from the implant lysates. DuoSet ELISA development systems for Mouse Neutrophil Elastase/ELA2 (DY4517), Mouse Myeloperoxidase (DY3667), and Mouse G-CSF (DY414) (R&D Systems, Inc., Minneapolis, MN, USA) were used according to the manufactured protocol. Briefly, 96-well microplates were coated overnight at room temperature with capture antibody at the working concentration (NE: 1 μg/mL, MPO: 800 ng/mL, G-CSF: 2 μg/mL). Then, the plates were washed three times with 1× wash buffer and blocked with 1% BSA in PBS reagent diluent for at least 1 h. After washing the blocking solution three times, sample and standards were added to each well and incubated for 2 h at room temperature. Wells were washed three times before the detection antibody was added at the working concentration (NE: 100 ng/mL, MPO: 20 ng/mL, G-CSF: 200 ng/mL) and incubated for 2 h at room temperature. Then, plates were washed again, and streptavidin-HRP (1:200 dilution) was added for 20 min limited from light, followed by another washing process and the addition of the substrate solution for 20 min. Reaction was stopped with 2N sulfuric acid and analyzed in a BioTek Synergy Neo2 multimode plate reader set at 450 nm with correction at 540 nm.

### Statistics

Treatment groups for each experiment were created by randomized mouse selection upon receipt at the facility. For comparisons between groups at specific time points, ordinary one-way analysis of variance (ANOVA) with Tukey’s correction for multiple comparisons was performed. Normality tests were not performed due to the small sample sizes. The sample size for each experiment is stated in the figure caption and ranges from *n* = 3 to *n* = 8. Data collection and analysis were not performed blind to the conditions of the experiments. *p*-values are shown in the figure captions and Supporting Information (Table S3).

## Supporting Information Available

Additional data for peptide purity and characterization, flow cytometry panel, histological data, SEM data, and statistical analyses (PDF)
