# Supplementary material for: Modulating Neutrophil Extracellular Trap Formation In Vivo with Locoregional Precision Using Differently Charged Self-Assembled Hydrogels
Source: ACS Cent Sci. 2025 Mar 12;11(3):465–78. doi: 10.1021/acscentsci.4c02198 (PMC11950866; doi:10.1021/acscentsci.4c02198)
Supplement: Supplementary file 1 — oc4c02198_si_001.pdf [file oc4c02198_si_001.pdf]

Modulating Neutrophil Extracellular Trap Formation In Vivo with Locoregional Precision using Differently Charged Self-Assembled Hydrogels

Tania L. Lopez-Silva, Caleb F. Anderson, Joel Schneider*

Chemical Biology Laboratory, Center for Cancer Research, National Cancer Institute, National Institutes of Health, Frederick, MD, 21702, United States.

SUPPORTING INFORMATION

Contents

Figure S1. RP-HPLC trace and ESI mass spectra 5
Figure S2. Temperature-dependent CD spectra of TLK5 6
Figure S3. Temperature-dependent CD spectra of TLE5 7
Figure S4. Time for gel stabilization 8
Figure S5. Rheological characterization of peptide gels 9
Figure S6. Tissue sections 10
Figure S7. Gating strategy for myeloid panel 11
Figure S8. H&E-stained tissue sections of TLE5 implants. 12
Figure S9. Monitoring hydrogel degradation using ultrasound imaging 13
Figure S10. Gross histology of subcutaneous TLK5 and TLE5 implants over time. 13
Figure S11. Peptide sequences of positively charged gel-forming peptides HLT2 and MAX8. 14
Figure S12. Characterization of the immune response to HLT2 and MAX8. 15
Figure S13. H&E-stained tissue sections of TLK5 implants 16
Figure S14. H&E-stained tissue sections of HLT2 implants 17
Figure S15. H&E-stained tissue sections of MAX8 implants 18
Figure S16. Representative immunofluorescence images of HLT2 and MAX8 19
Figure S17. Representative immunofluorescence images of HLT2 and MAX8 20
Figure S18. Representative immunofluorescence images of different implants 21
Figure S19. Representative immunofluorescence images of different implants 22
Figure S20. SEM images of TLK5 and TLE5 gels 23
Figure S21. Immunofluorescence staining of TLE5 implants 14- and 30-days post-injection. 24
Figure S22. H&E-stained tissue sections of the gastrocnemius muscle with gel injections. 24
Figure S23. H&E-stained tissue sections of muscle near the gel implant. 24
Figure S24. Appearance of gel composites with different TLK5 content. 25
Table S1. Primary and secondary antibodies for immunofluorescence staining 25
Table S2. Flow cytometry panel for identifying infiltrating cells 26
Table S3. Statistical comparisons for Figure 5- Enzymes, cytokines, and chemokines. 26
Neutrophil Elastase (NE) Day 1 26
Neutrophil Elastase (NE) Day 3 26
Neutrophil Elastase (NE) Day 7 26
Neutrophil Elastase (NE) Day 14 27
Myeloperoxidase (MPO) Day 1 27

Myeloperoxidase (MPO) Day 3 27
Myeloperoxidase (MPO) Day 7 27
Myeloperoxidase (MPO) Day 14 27
CXCL1 (KC) Day 1 27
CXCL1 (KC) Day 3 27
CXCL1 (KC) Day 7 28
CXCL1 (KC) Day 14 28
CCL3 (MIP-1α) Day 1 28
CCL3 (MIP-1α) Day 3 28
CCL3 (MIP-1α) Day 7 28
CCL3 (MIP-1α) Day 14 28
CCL2 (MCP-1) Day 1 29
CCL2 (MCP-1) Day 3 29
CCL2 (MCP-1) Day 7 29
CCL2 (MCP-1) Day 14 29
IL-6 Day 1 29
IL-6 Day 3 30
IL-6 Day 7 30
IL-6 Day 14 30
TNF-α Day 1 30
TNF-α Day 3 30
TNF-α Day 7 30
TNF-α Day 14 30
IL-1β Day 1 31
IL-1β Day 3 31
IL-1β Day 7 31
IL-1β Day 14 31
G-CSF Day 1 31
G-CSF Day 3 32
G-CSF Day 7 32
G-CSF Day 14 32
CCL5 RANTES Day 1 32
CCL5 RANTES Day 3 32
CCL5 RANTES Day 7 32
CCL5 RANTES Day 14 33

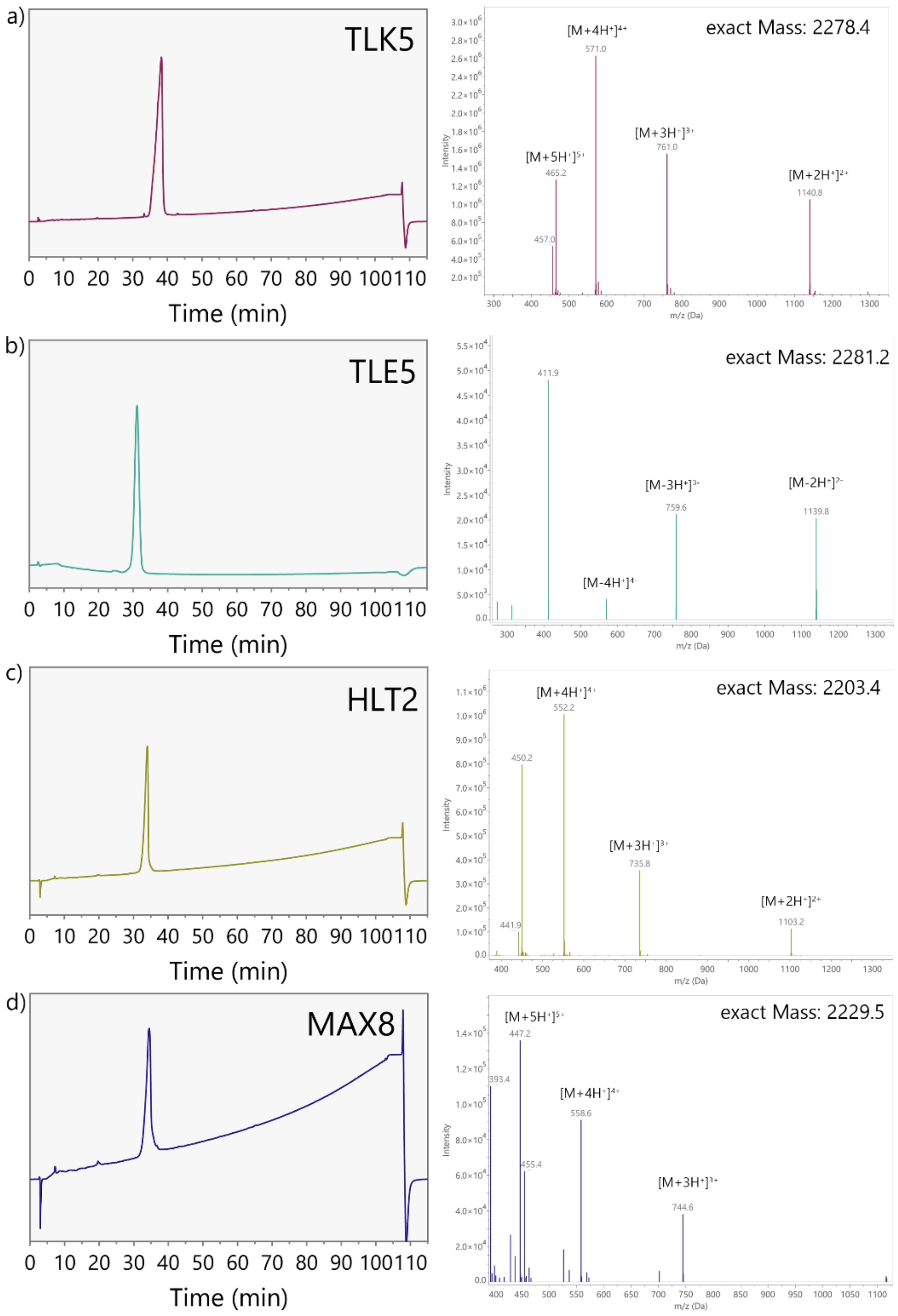

Figure S1. RP-HPLC trace and ESI mass spectra of a) TLK5, tR = 38 min, calculated mass: $[M+H^+]^{1+}$ = 2279.4, $[M+2H^+]^{2+}$ = 1140.2, $[M+3H^+]^{3+}$ = 760.5, $[M+4H^+]^{4}$ = 570.6, $[M+5H^+]^{5+}$ = 456.7. b) TLE5, tR = 31 min, calculated mass: $[M-H^+]^{1-}$ = 2280.2, $[M-2H^+]^{2-}$ = 1139.6, $[M-3H^+]^{3-}$ = 759.4, $[M-4H^+]^{4-}$ = 569.3, $[M-5H^+]^{5-}$ = 455.2. c) HLT2, tR = 34 min, calculated mass: $[M+H^+]^{1+}$ = 2204.4, $[M+2H^+]^{2+}$ = 1102.7, $[M+3H^+]^{3+}$ = 735.5, $[M+4H^+]^{4}$ = 551.9, $[M+5H^+]^{5+}$ = 441.7. d) MAX8, tR = 36 min, calculated mass: $[M+H^+]^{1+}$ = 2230.5, $[M+2H^+]^{2+}$ = 1115.8, $[M+3H^+]^{3+}$ = 744.2, $[M+4H^+]^{4}$ = 558.4, $[M+5H^+]^{5+}$ = 446.9.

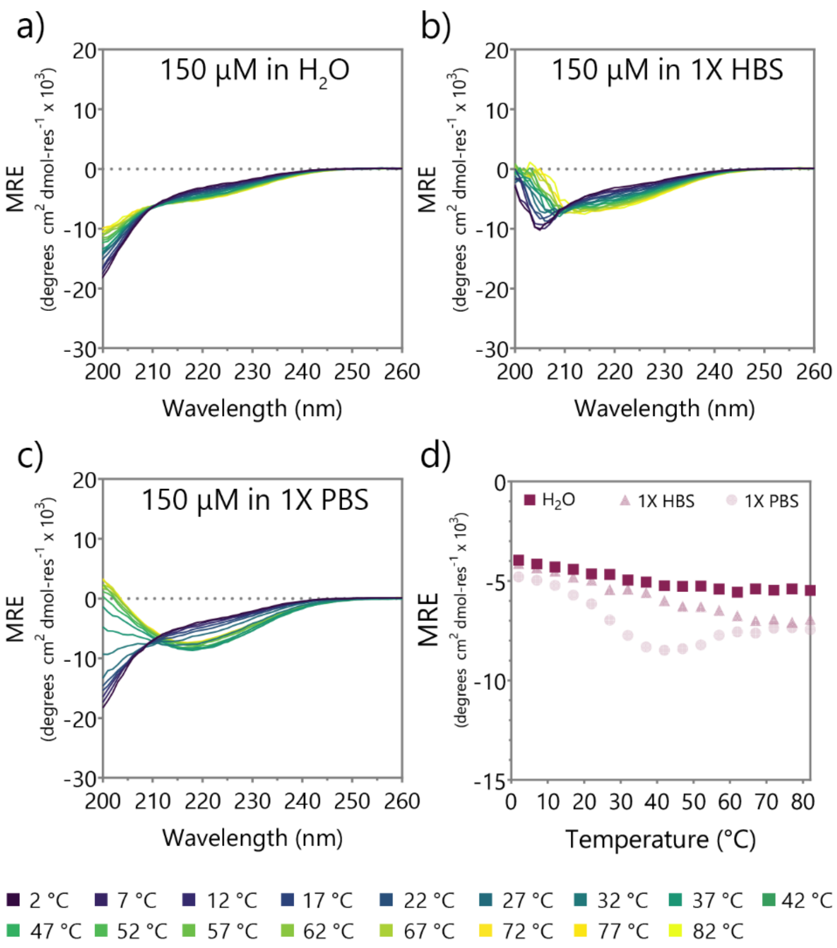

Figure S2. Temperature-dependent CD spectra of TLK5 at 150 μM in a) water, b) 1X HBS pH 7.4, and c) 1X PBS pH 7.4. d) MRE values at 216 nm as a function of temperature to monitor β-sheet formation. At this concentration, TLK5 does not form a β-sheet and assemble. In 1X PBS, TLK5 forms a β-sheet with the characteristic minimum at 216 nm but precipitates out of the solution.

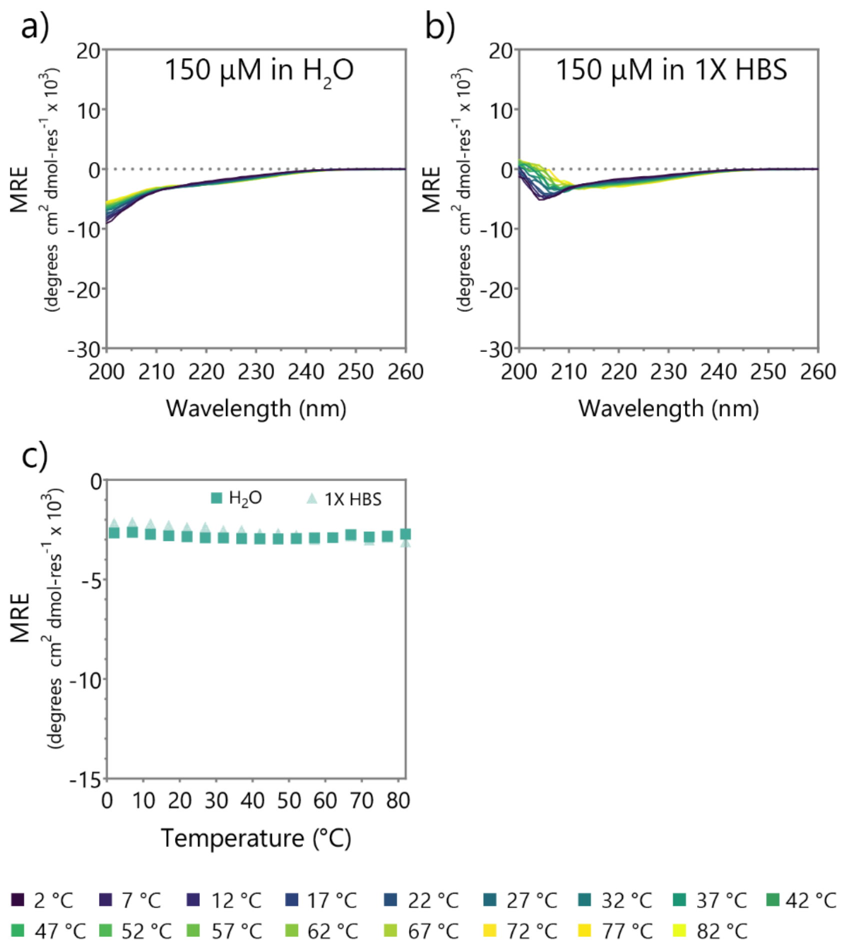

Figure S3. Temperature-dependent CD spectra of TLE5 at 150 µM in a) water and b) 1X HBS pH 7.4. d) MRE values at 216 nm as a function of temperature to monitor β-sheet formation. At this concentration, TLE5 does not form a β-sheet and assemble.

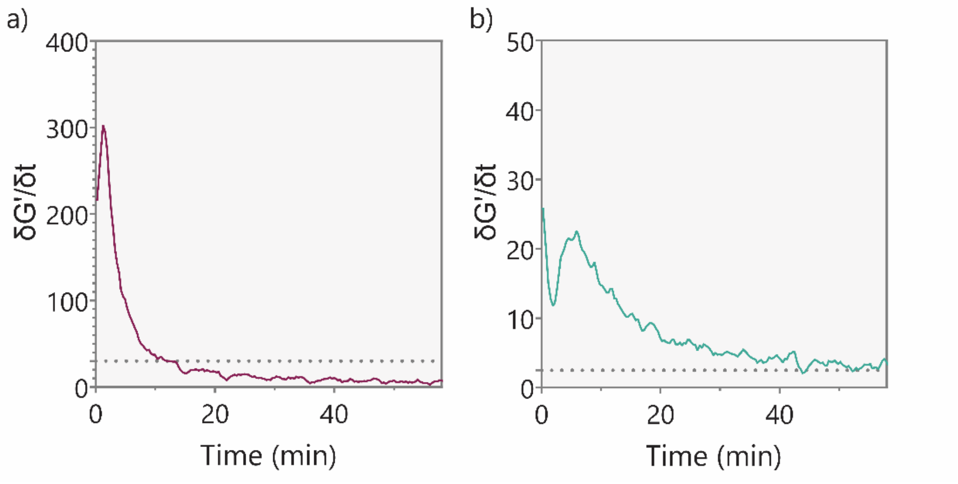

Figure S4. Time for gel stabilization for a) TLK5 and b) TLE5 gels. First derivative of the averaged storage modulus vs time represents the change in G' as a function of time. Time of stabilization was determined as ≤10% of change in G'.

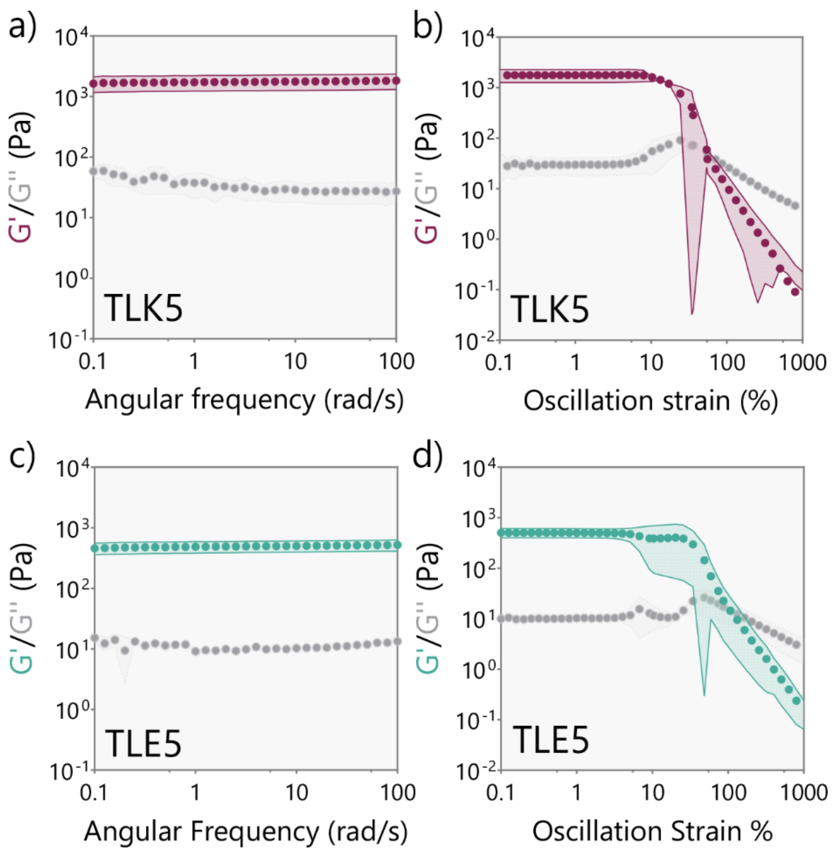

Figure S5. Rheological characterization of peptide gels. Frequency sweep (a) and amplitude sweep (b) of TLK5 1 wt.% gel. Frequency sweep (c) and amplitude sweep (d) of TLE5 1 wt.% gel. Data are shown as mean and standard deviation (with error bands) n = 3.

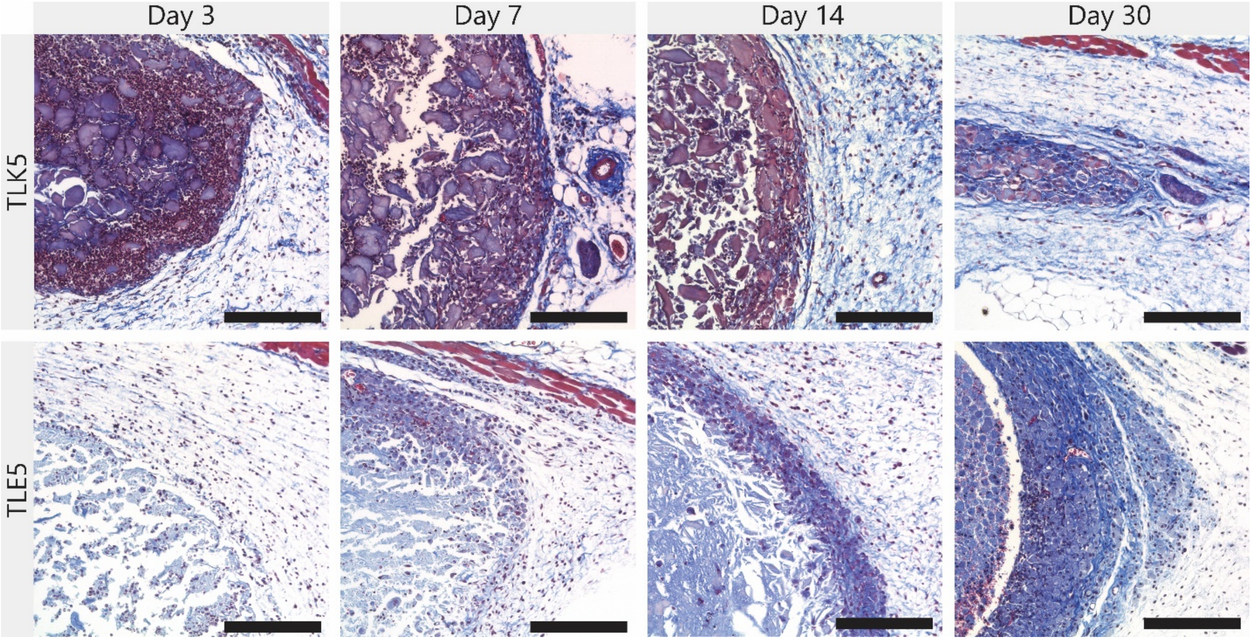

Figure S6. Tissue sections of the different hydrogel implants in the subcutaneous space stained with Masson's Trichrome at different time points. Scale bar = 200 μm.

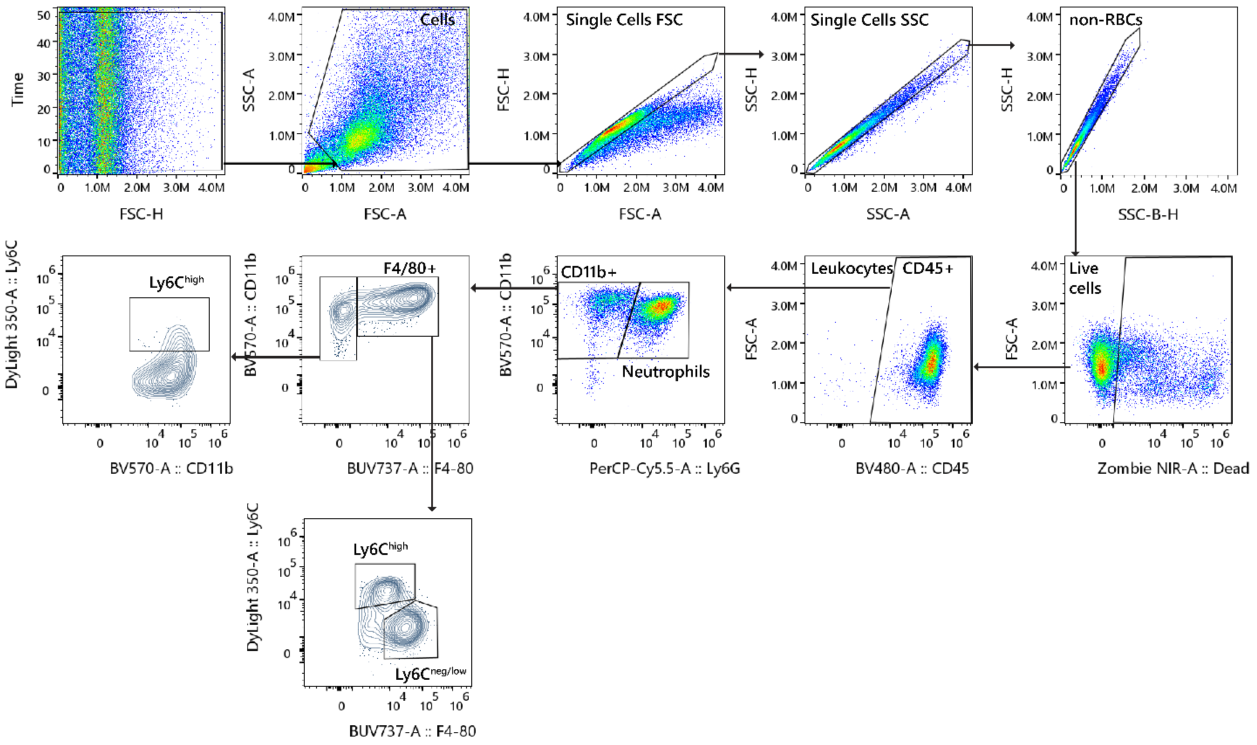

Figure S7. Gating strategy for myeloid panel to identify Neutrophils (CD45+CD11b+Ly6G+), and macrophages (CD45+CD11b+F4/80+Ly6Chigh or low/neg). Data was cleaned by gating on steady flow, viable cells, singlets, non-red blood cells, and live cells.

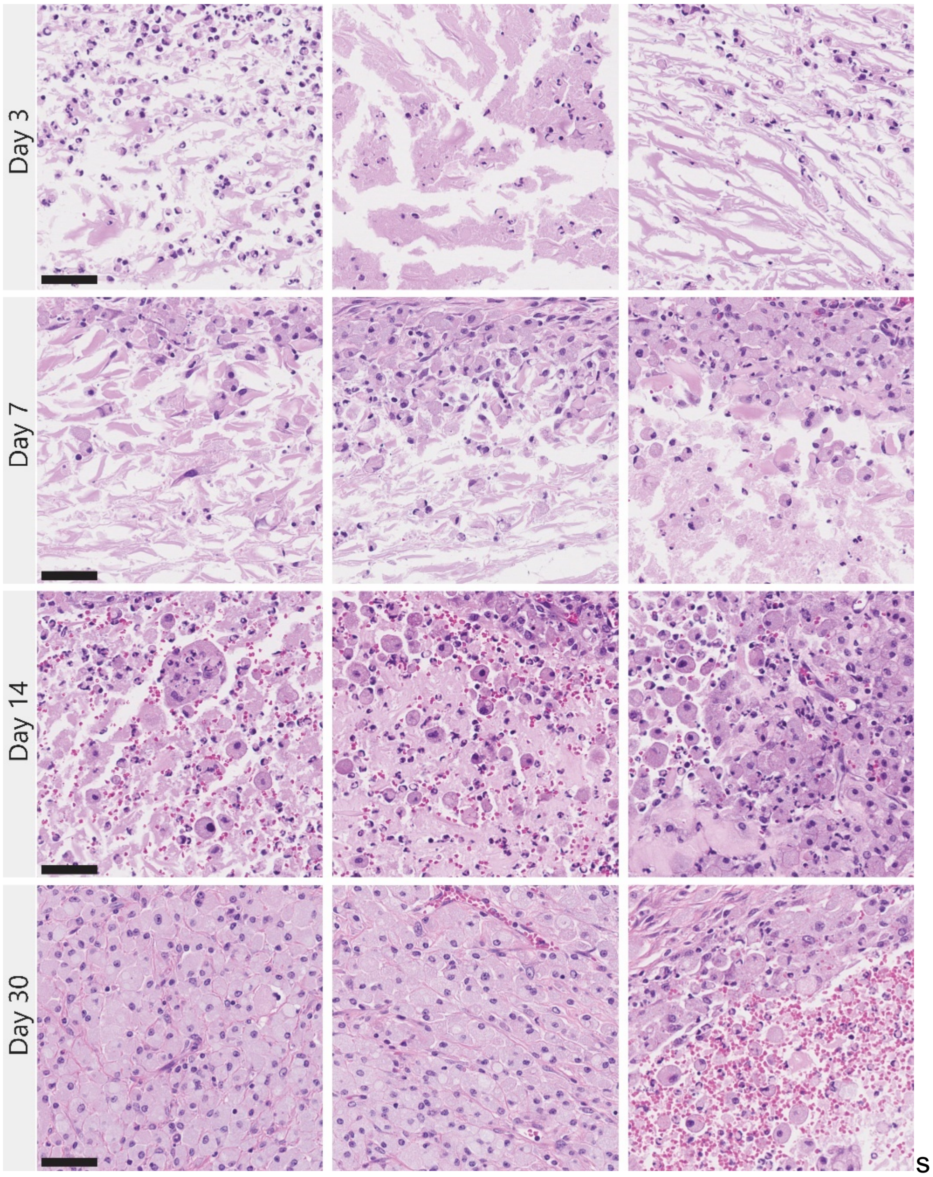

Figure S8. H&E-stained tissue sections of TLE5 implants. Hydrogel presents a lower degree of cellular infiltration, mostly in the periphery and surface of the implants. At later time points, there is more dense cell infiltration in the periphery and the core contains red blood cells, neutrophils, and macrophages. Scale bar = 50 μm.

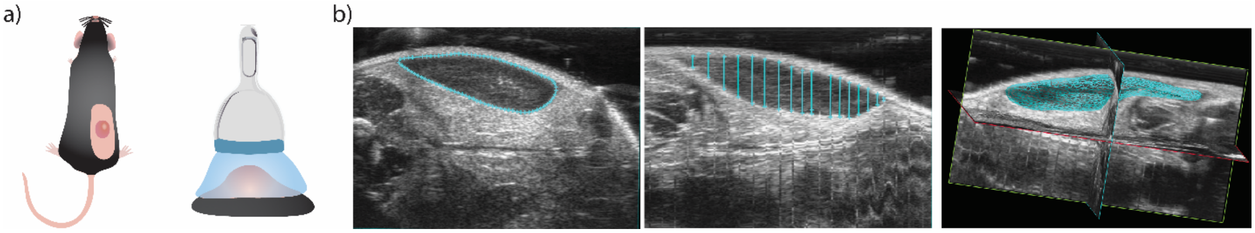

Figure S9. Monitoring hydrogel degradation using ultrasound imaging. a) Mice were subcutaneously injected with 150 µL hydrogels in the dorsal flank and the implant volume was obtained with ultrasound imaging. b) Examples of ultrasound images and volume determination using Vevo LAB software.

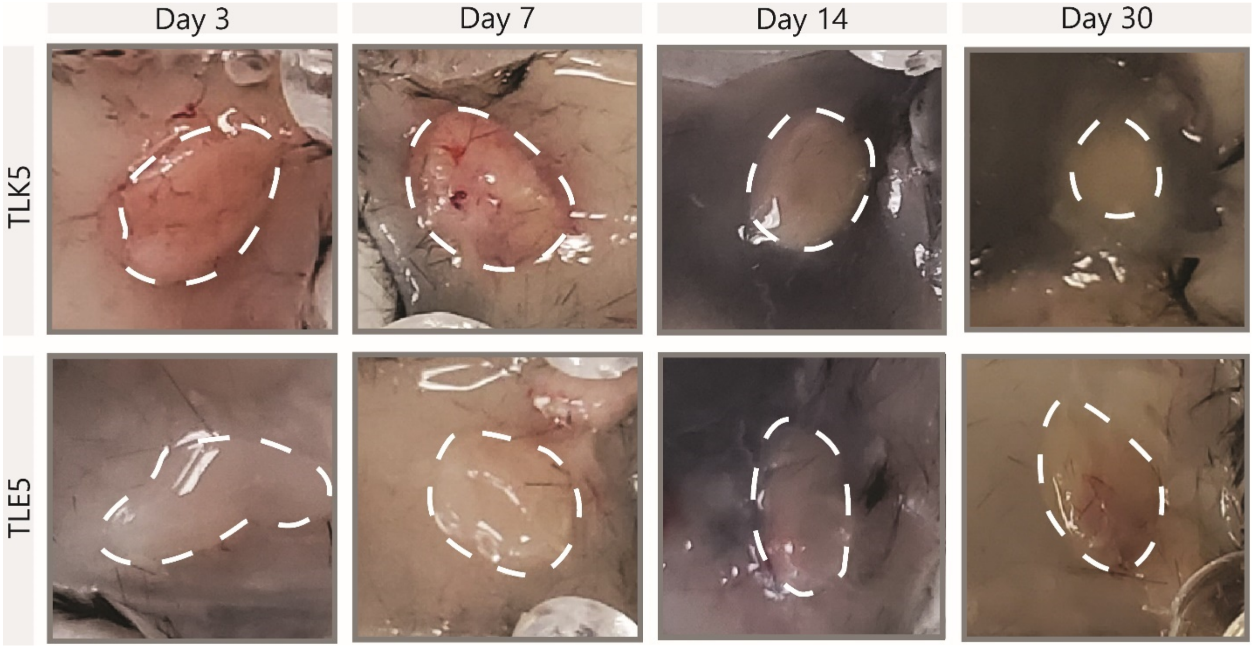

Figure S10. Gross histology of subcutaneous TLK5 and TLE5 implants over time.

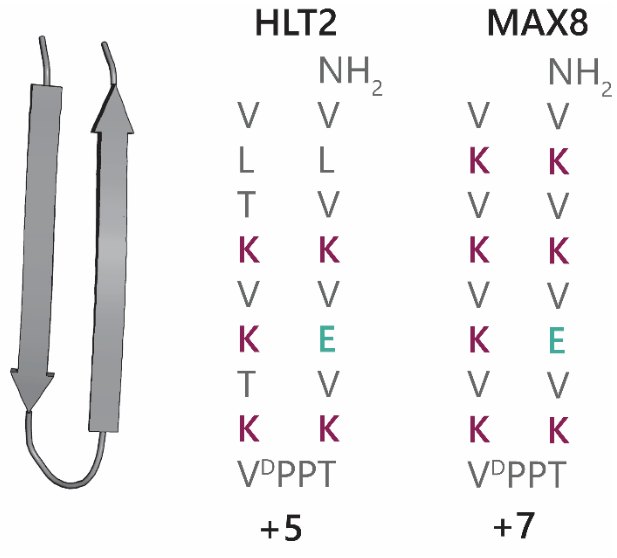

Figure S11. Peptide sequences of positively charged gel-forming peptides HLT2 and MAX8.

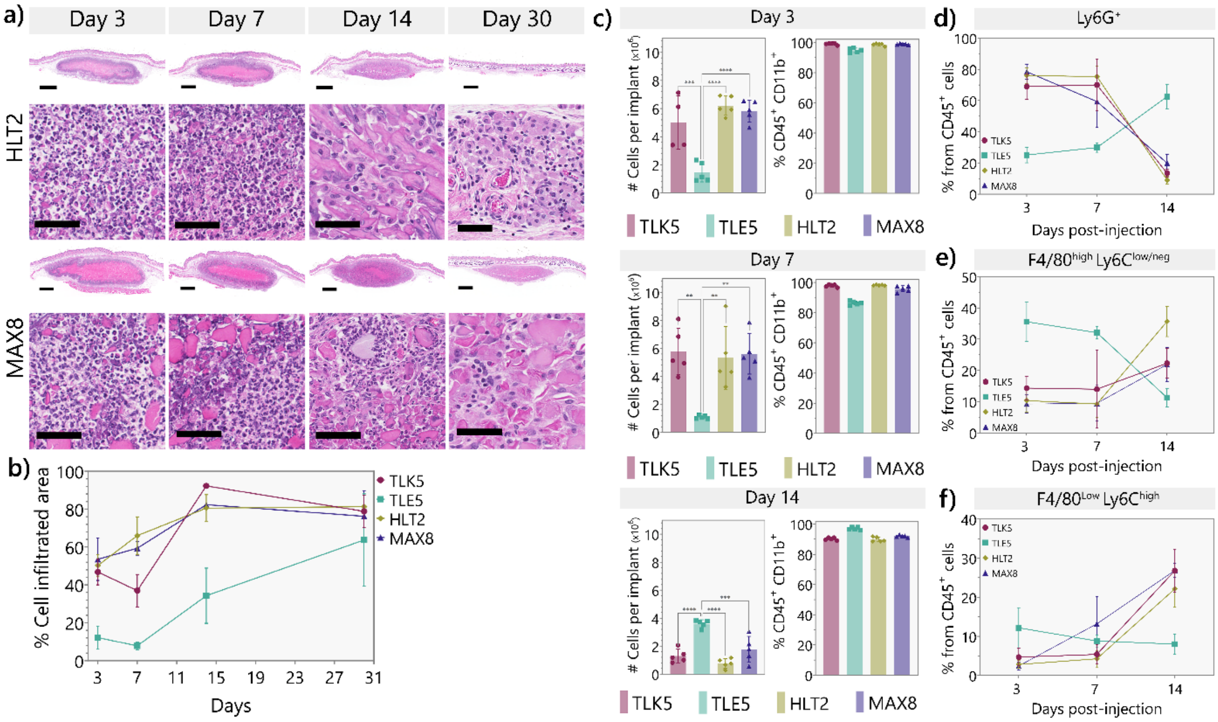

Figure S12. Characterization of the immune response to HLT2 and MAX8. **a)** H&E-stained tissue sections of implants for HLT2 and MAX8 gels at 3-, 7-, 14-, and 30- days post-injection. **b)** Percentage of infiltrated area over time determined by histology, n = 3 mice. *Statistical differences at day 3: TLE5 vs TLK5, HLT2 ** p-value < 0.003, TLE5 vs MAX8 *** p-value 0.0008; at day 7: TLE5 vs TLK5 ** p-value 0.0037, TLE5 vs MAX8, HLT2 **** p-value <0.0001, at day 14: TLE5 vs HLT2, MAX8 ** p-value < 0.003, TLE5 vs TLK5 *** p-value 0.0004.* **c)** Number of cells recovered per implant and % myeloid cells ($CD45^{+}CD11b^{+}$) at day 3, day 7, and day 14 post-injection. n = 5 mice. **d)** Percentage of $CD45^{+}CD11b^{+}Ly6G^{+}$ cells (Neutrophils) from total leukocytes for each peptide hydrogel at different timepoints. n = 5 mice. *Statistical comparison: day 3 TLK5, HLT2, MAX8 vs. TLE5 p-value < 0.0001; day 7 TLK5, HLT2 vs. TLE5 p-value < 0.0005, TLE5 vs. MAX8 p-value 0.0075; day 14 TLK5, HLT2, MAX8 vs. TLE5 p-value <0.0001, HLT2 vs. MAX8 p-value 0.0193.* **e)** Percentage of $CD45^{+}CD11b^{+}Ly6G^{neg}F4/80^{high}Ly6C^{low/neg}$ cells from total $CD45^{+}$ leukocytes for each peptide hydrogel at different timepoints. *Statistical comparison: Day 3, TLK5, HLT2, MAX8 vs. TLE5 p-value < 0.0001. Day 7, TLK5 vs. TLE5 p-value 0.0057, TLE5 vs. HLT2 and MAX8 p-value < 0.0008. Day 14 TLK5 vs. TLE5 and HLT2 p-value <0.0080, TLE5 vs. HLT2 p-value < 0.0001, TLE5 vs MAX8 p-value 0.0103, HLT2 vs. MAX8 p-value 0.0012.* **f)** Percentage of $CD45^{+}CD11b^{+}Ly6G^{neg}F4/80^{low}Ly6C^{high}$ cells (monocytes/macrophages) from total $CD45^{+}$ leukocytes for each peptide hydrogel at 3, 7, and 14 days-post injection. *Statistical comparison: Day 3, TLK5 vs TLE5 p-value 0.0044, TLE5 vs HLT2 and MAX8 p-value <0.0006. Day 7, MAX8 vs TLK5 and HLT2 p-value < 0.0296. Day 14, TLE5 vs TLK5 and MAX8 p-value < 0.0001, TLE5 vs HLT2 p-value 0.0002.* n = 5 mice. Error bars represent standard deviation.

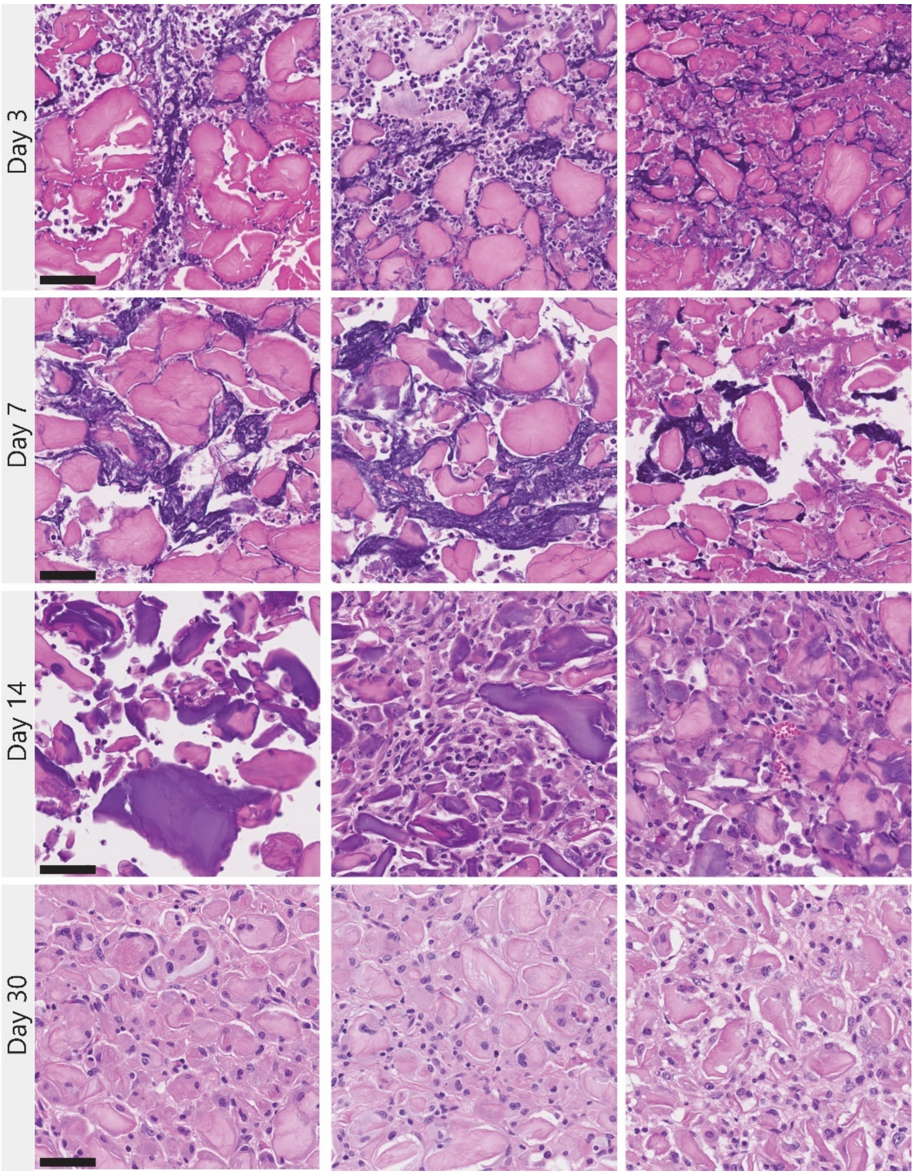

Figure S13. H&E-stained tissue sections of TLK5 implants at different time points. At day 3 and 7 post-injections, the implants present areas with extracellular traps seen as basophilic DNA fibrous networks. Scale bar = 50 μm.

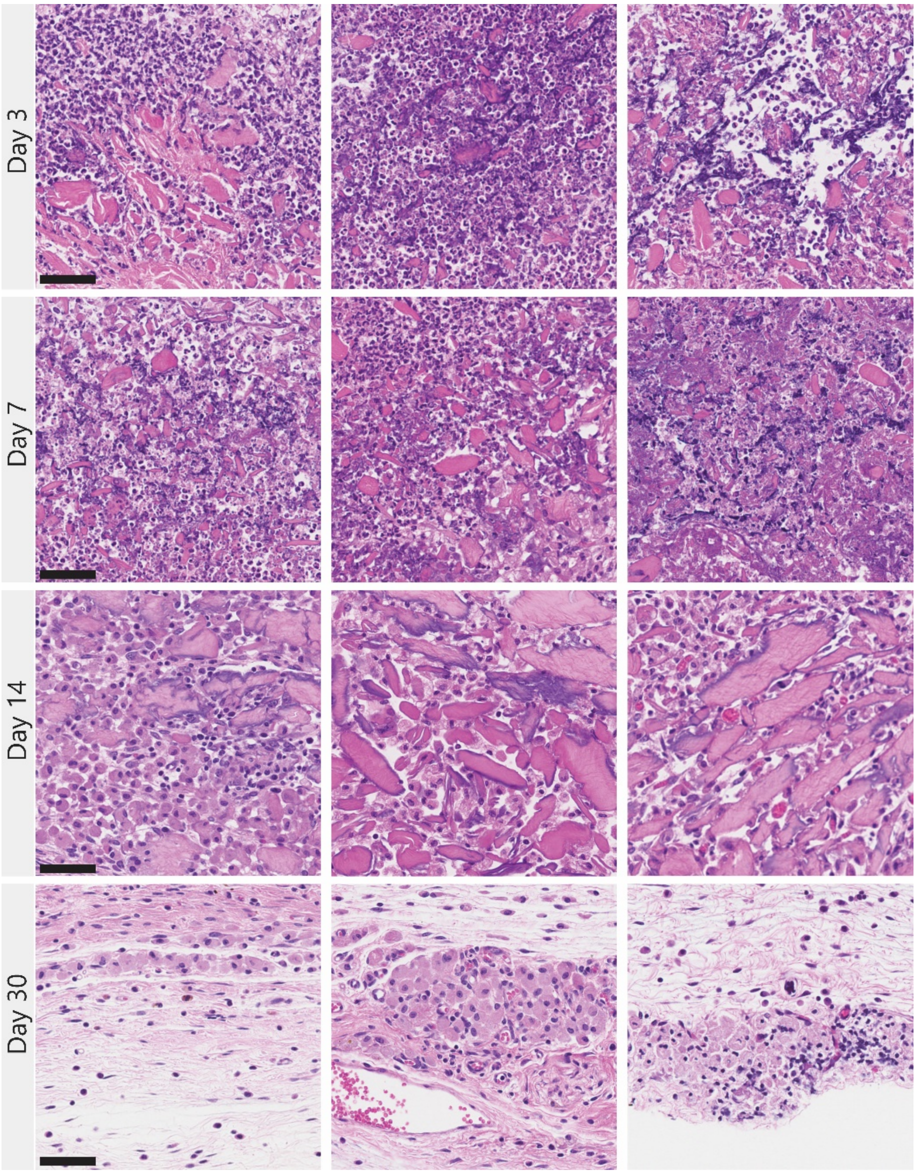

Figure S14. H&E-stained tissue sections of HLT2 implants at different time points. At day 3 and 7 post-injections, the implants present areas with extracellular traps seen as basophilic DNA fibrous networks. Scale bar = 50 μm.

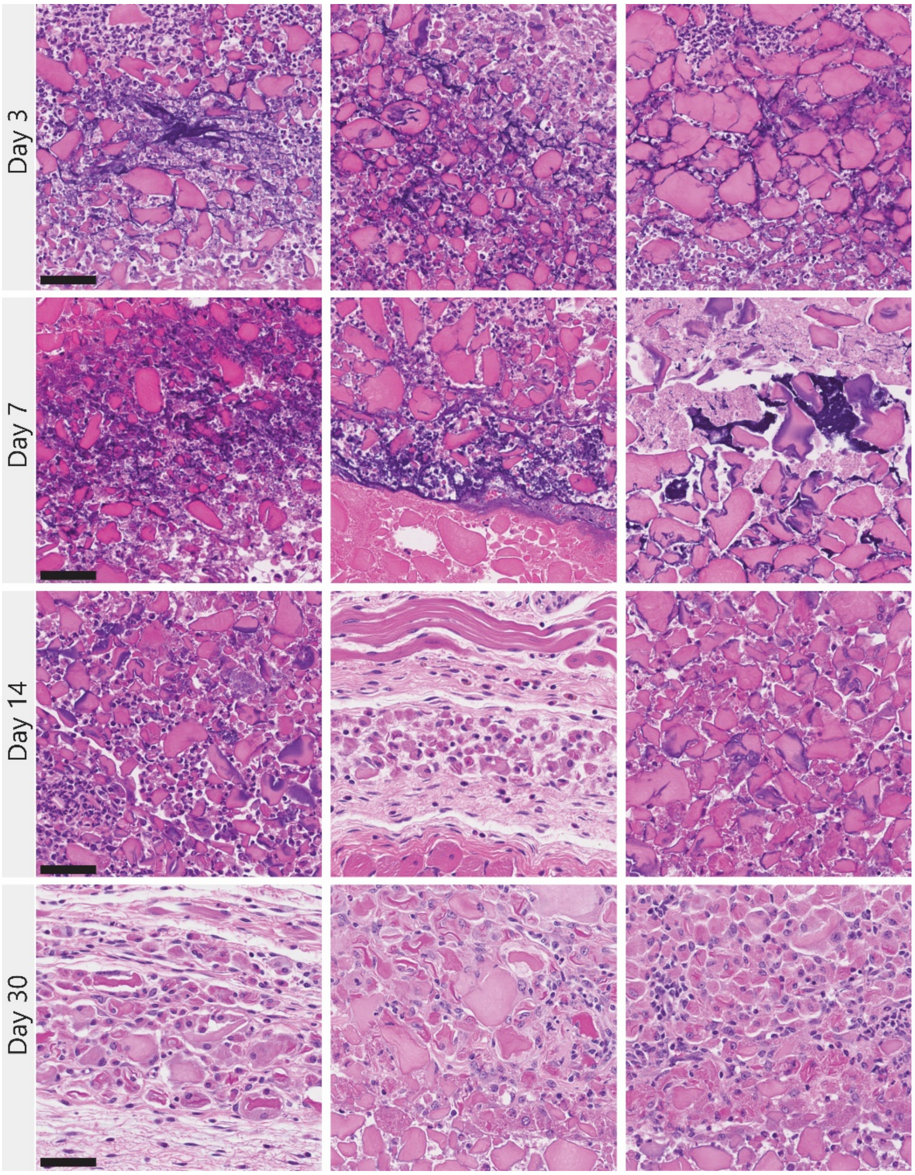

Figure S15. H&E-stained tissue sections of MAX8 implants at different time points. At day 3 and 7 post-injections, the implants present areas with extracellular traps seen as basophilic DNA fibrous networks. Scale bar = 50 μm.

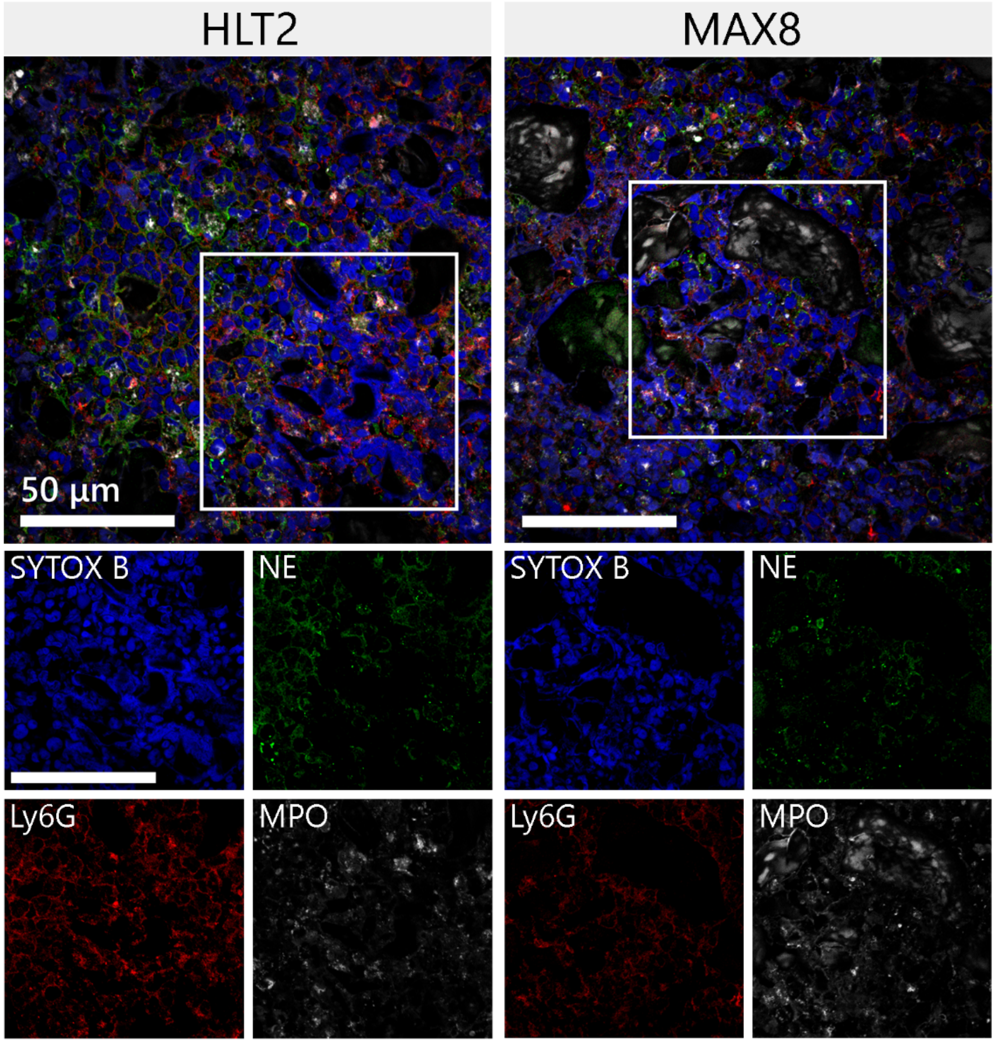

Figure S16. Representative immunofluorescence images of HLT2 and MAX8 implants stained for NETs markers NE (green), MPO (gray), DNA (blue), and Ly6G (red) at day 3 post injection. Scale bar = 50 µm.

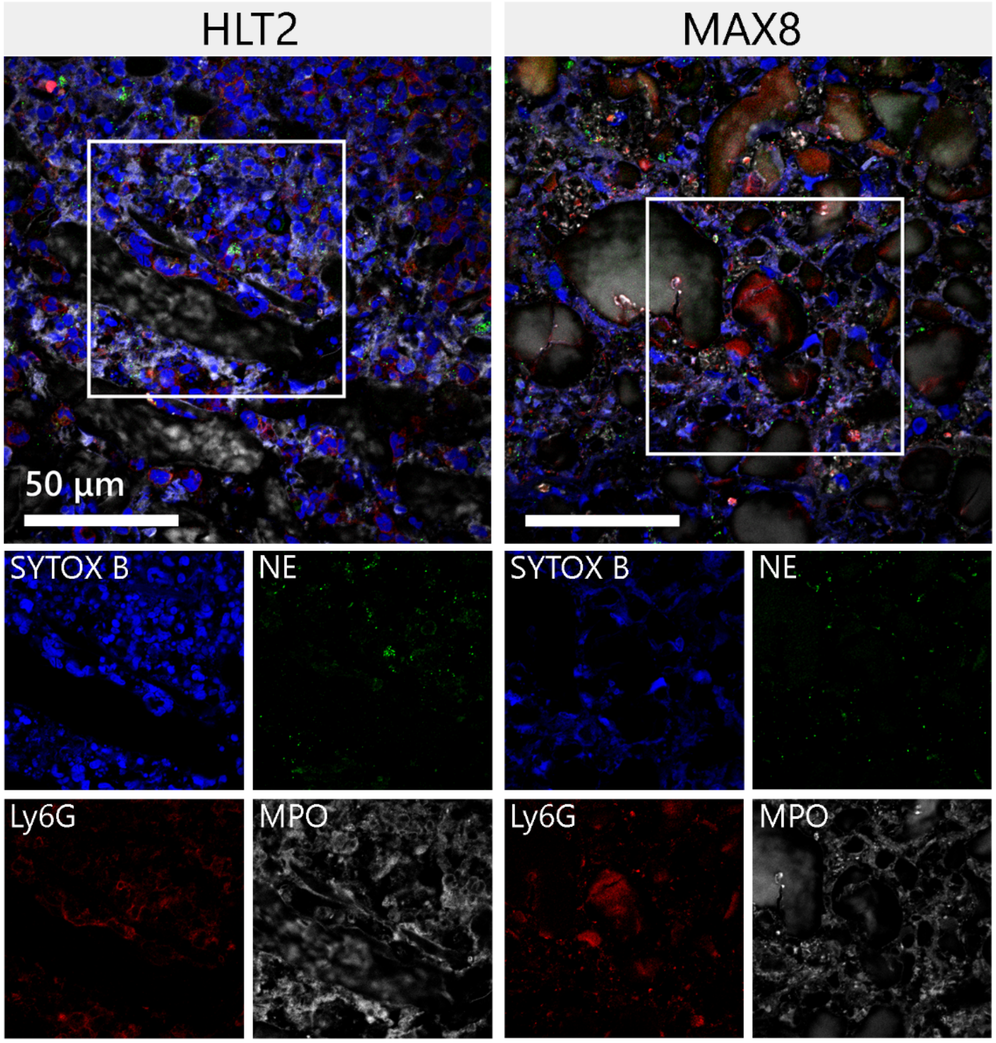

Figure S17. Representative immunofluorescence images of HLT2 and MAX8 implants stained for NETs markers NE (green), MPO (gray), DNA (blue), and Ly6G (red) at day 7 post injection. Scale bar = 50 µm.

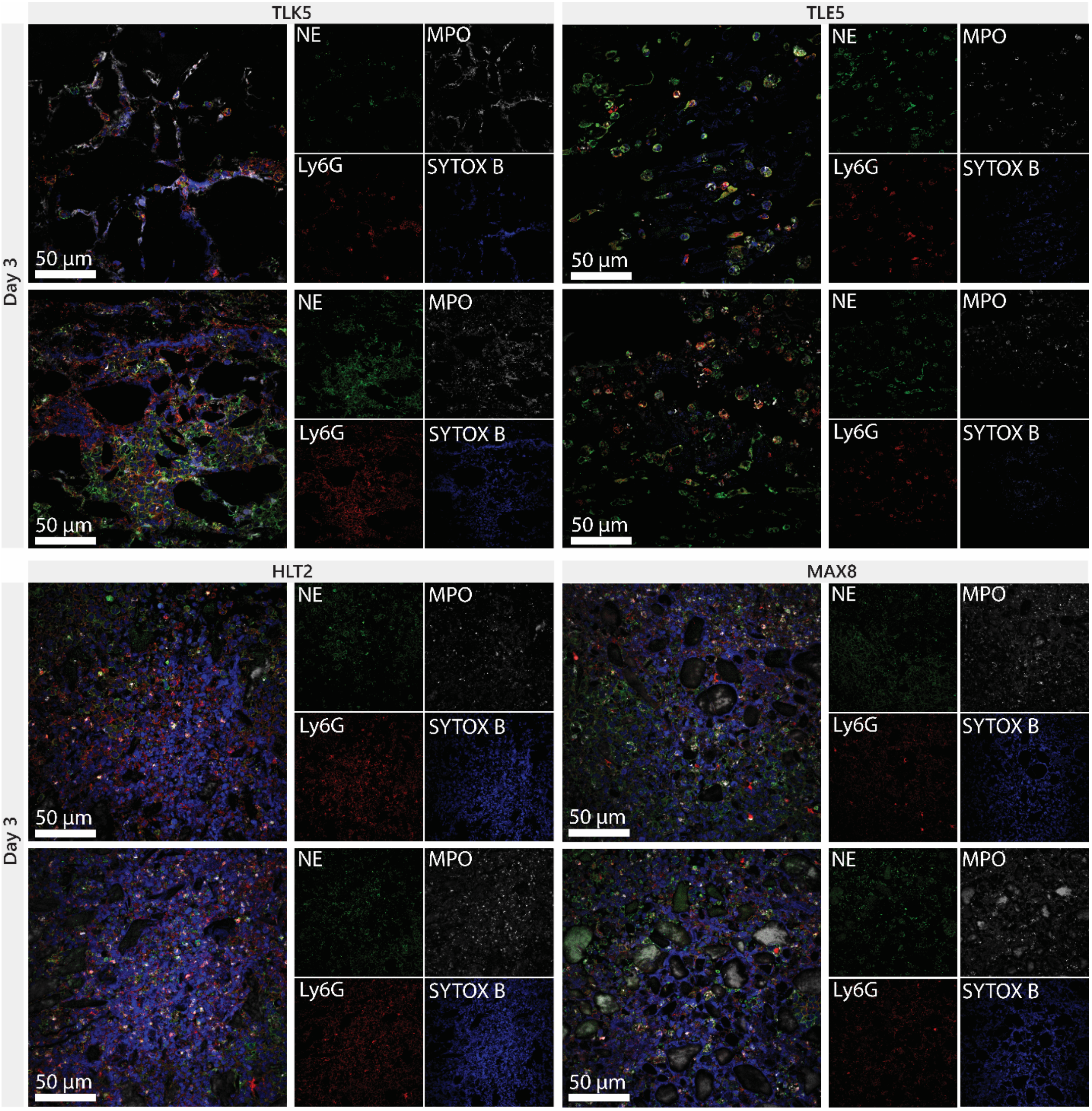

Figure S18. Representative immunofluorescence images of different implants for TLK5, TLE5, HLT2, and MAX8 hydrogels three days post-injection.

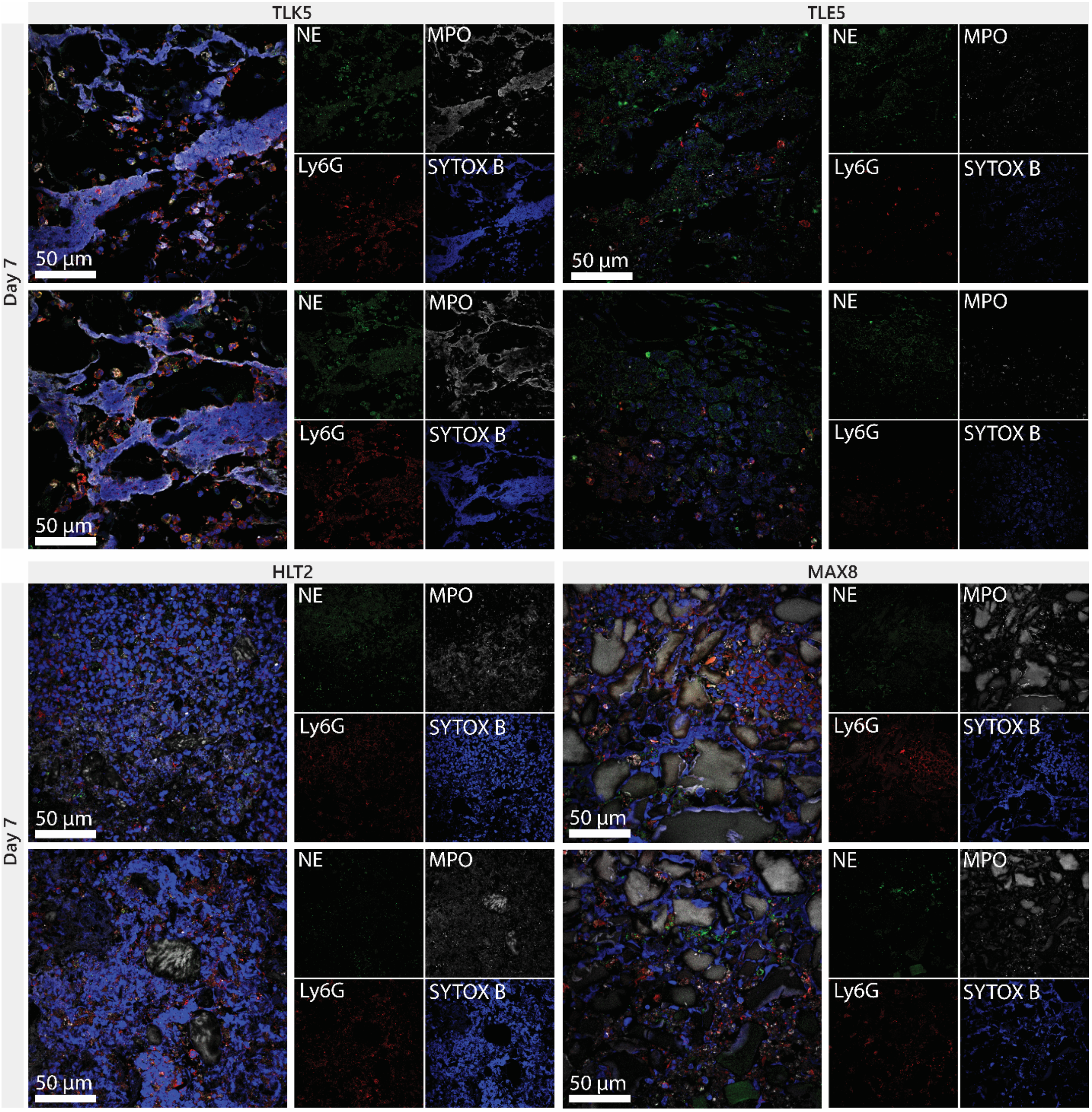

Figure S19. Representative immunofluorescence images of different implants for TLK5, TLE5, HLT2, and MAX8 hydrogels seven days post-injection.

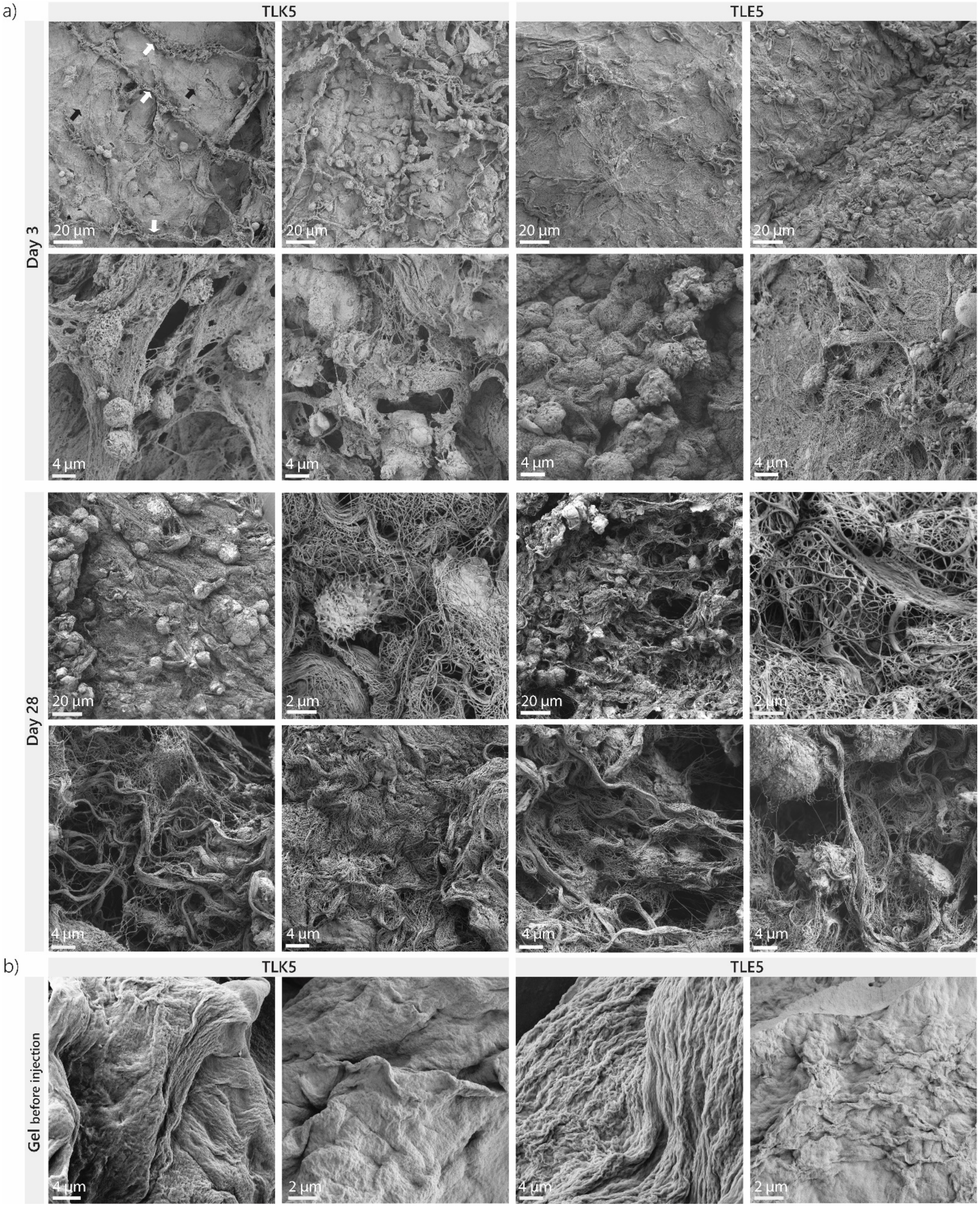

Figure S20. SEM images of TLK5 and TLE5 gels. a) TLK5 and TLE5 implants injected in the subcutaneous space at day 3 and day 28 post-injection. TLK5 gel is shown as black arrows and NETs are shown as white arrows. b) SEM images of TLK5 and TLE5 native hydrogels before injection.

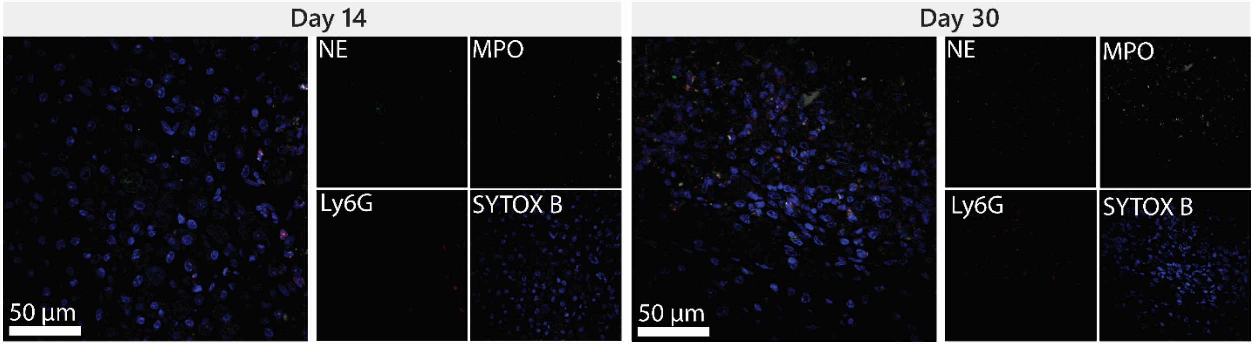

Figure S21. Immunofluorescence staining of TLE5 implants 14- and 30-days post-injection.

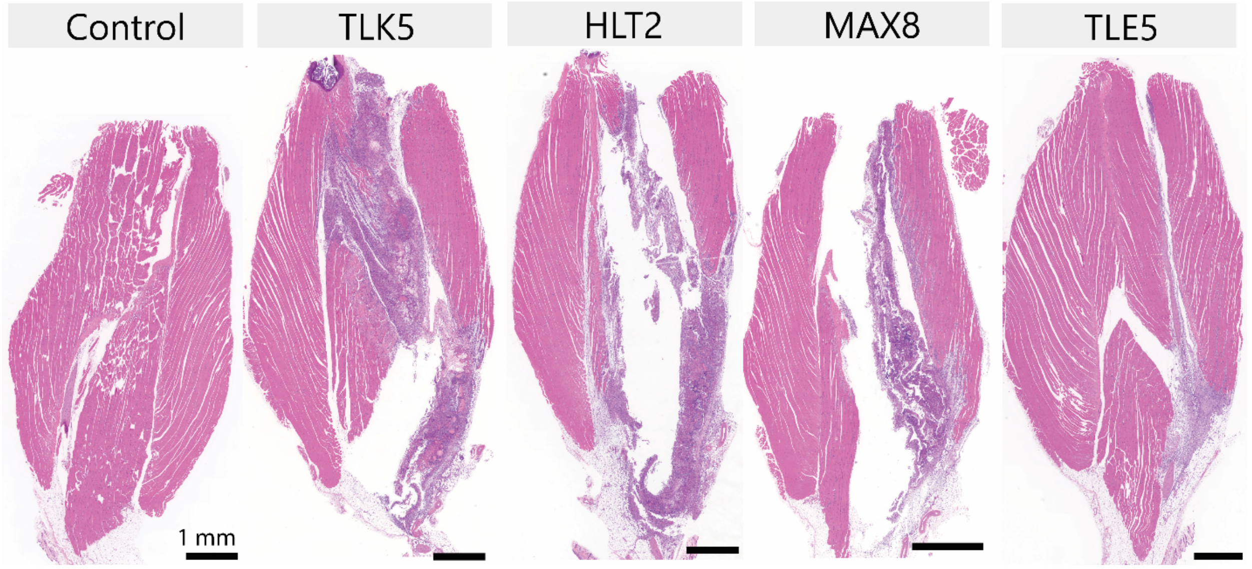

Figure S22. H&E-stained tissue sections of the gastrocnemius muscle with gel injections.

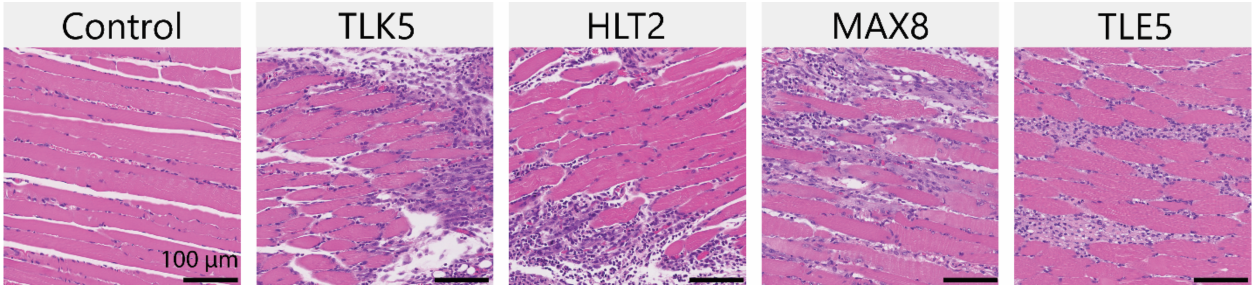

Figure S23. H&E-stained tissue sections of muscle near the gel implant.

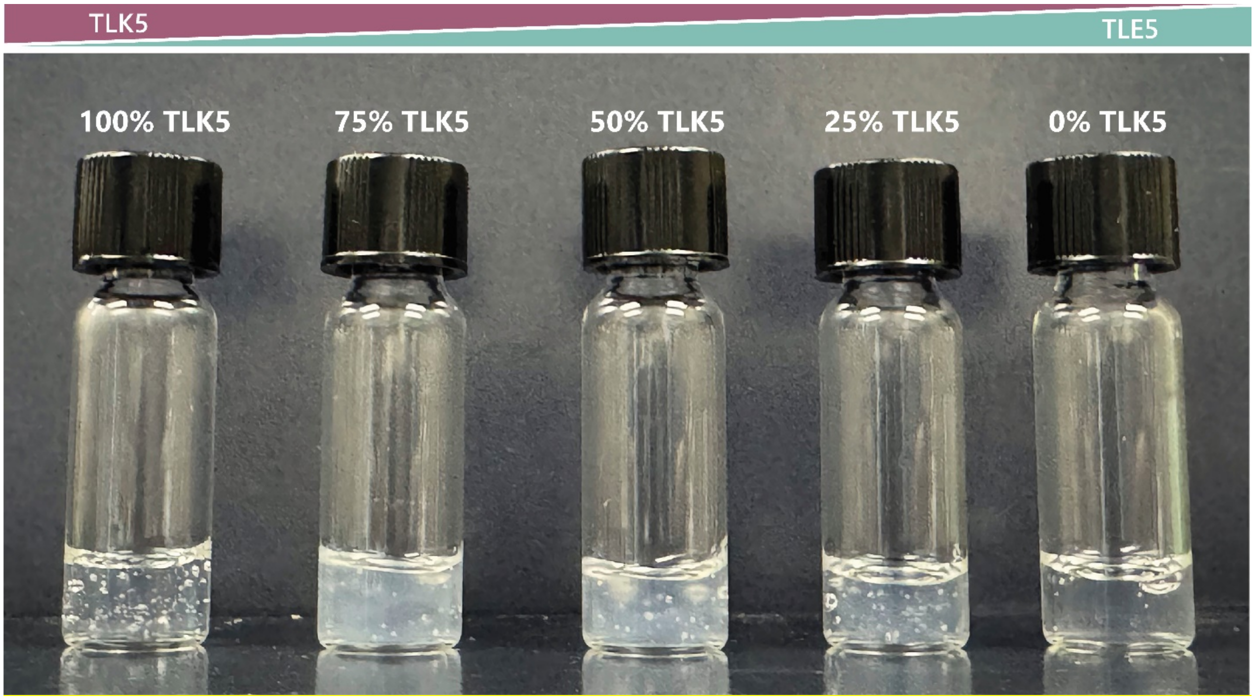

Figure S24. Appearance of gel composites with different TLK5 content.

Table S1. *Primary and secondary antibodies for immunofluorescence staining*

Specificity	Reactivity	Fluorochrome	Isotype	Clone	Vendor Cat #	Concentration	Dilution
Primary Antibodies							
Ly6G	Mouse	-	Rat IgG2a, κ	1A8	Biolegend 127602	0.5 mg/mL	1:500
Neutrophil Elastase	Mouse, Human	-	Rabbit IgG	JF098-6	Novus NBP2-66972	1 mg/mL	1:200
Myeloperoxidase	Mouse Human	-	Goat IgG	Polyclonal	R&D systems AF3667	0.2 mg/mL	1:100
SYTOX™ Blue (DNA)	-	SYTOX™ Blue	-	-	Thermo Fisher Scientific S11348	5 mM	1:1000
Secondary Antibodies							
Goat IgG	Goat	Alexa Fluor™ 647	Donkey IgG	Polyclonal	Thermo Fisher Scientific A21447	2 mg/mL	1:500

Rat IgG	Rat	Alexa Fluor™ 568	Donkey IgG	Polyclonal	Thermo Fisher Scientific A78946	2 mg/mL	1:500
Rabbit IgG	Rabbit	Alexa Fluor™ 488	Donkey IgG	Polyclonal	Thermo Fisher Scientific A21206	2 mg/mL	1:500

Table S2. *Flow cytometry panel for identifying infiltrating cells*

Specificity	Fluorochrome	Clone	Isotype	Vendor	Catalog #	Titer (ng/test)
Viability	Zombie NIR			Biolegend	423106	100 µL of 1:2000 dilution from stock
CD45	BV480	30-F11	Rat LOU/M IgG2b, κ	BD	566168	100
CD11b	BV570	M1/70	Rat IgG2b, κ	Biolegend	101233	200
Ly6G	PerCP-Cy5.5	1A8	Rat Lewis IgG2a, κ	BD	560602	400
F4/80	BUV737	T45-2342	Rat WI IgG2a, κ	BD	749283	400
Ly6C	Dylight350	ER-MP20	Rat IgG2a	Novus Biologicals	NB100-65413UV	710

Table S3. Statistical comparisons for Figure 5- Enzymes, cytokines, and chemokines.

Neutrophil Elastase (NE) Day 1

Tukey's multiple comparisons test	Mean Diff.	95.00% CI of diff.	Below threshold?	Summary	Adjusted P Value
TLK5 vs. HLT2	-13883	-33597 to 5832	No	ns	0.2236
TLK5 vs. MAX8	-11855	-31570 to 7859	No	ns	0.3458
TLK5 vs. TLE5	23674	3960 to 43389	Yes	*	0.0161
HLT2 vs. MAX8	2027	-17687 to 21742	No	ns	0.9908
HLT2 vs. TLE5	37557	17843 to 57271	Yes	***	0.0003
MAX8 vs. TLE5	35530	15815 to 55244	Yes	***	0.0005

Neutrophil Elastase (NE) Day 3

Tukey's multiple comparisons test	Mean Diff.	95.00% CI of diff.	Below threshold?	Summary	Adjusted P Value
TLK5 vs. HLT2	-3603	-20112 to 12906	No	ns	0.9227
TLK5 vs. MAX8	4620	-11889 to 21129	No	ns	0.8531
TLK5 vs. TLE5	16015	-494.5 to 32524	No	ns	0.0589
HLT2 vs. MAX8	8223	-8286 to 24732	No	ns	0.5027
HLT2 vs. TLE5	19618	3109 to 36127	Yes	*	0.0173
MAX8 vs. TLE5	11395	-5114 to 27904	No	ns	0.2381

Neutrophil Elastase (NE) Day 7

Tukey's multiple comparisons test	Mean Diff.	95.00% CI of diff.	Below threshold?	Summary	Adjusted P Value
TLK5 vs. HLT2	3878	-1442 to 9198	No	ns	0.1997
TLK5 vs. MAX8	4928	-392.3 to 10247	No	ns	0.0745
TLK5 vs. TLE5	12887	7567 to 18207	Yes	****	<0.0001
HLT2 vs. MAX8	1050	-4270 to 6369	No	ns	0.9412
HLT2 vs. TLE5	9009	3689 to 14329	Yes	***	0.0009
MAX8 vs. TLE5	7959	2639 to 13279	Yes	**	0.0029

Neutrophil Elastase (NE) Day 14

Tukey's multiple comparisons test	Mean Diff.	95.00% CI of diff.	Below threshold?	Summary	Adjusted P Value
TLK5 vs. HLT2	877.2	-2268 to 4022	No	ns	0.8543
TLK5 vs. MAX8	-6268	-9413 to -3124	Yes	***	0.0002
TLK5 vs. TLE5	-3293	-6438 to -148.6	Yes	*	0.0385
HLT2 vs. MAX8	-7146	-10290 to -4001	Yes	****	<0.0001
HLT2 vs. TLE5	-4171	-7315 to -1026	Yes	**	0.0078
MAX8 vs. TLE5	2975	-169.7 to 6120	No	ns	0.0670

Myeloperoxidase (MPO) Day 1

Tukey's multiple comparisons test	Mean Diff.	95.00% CI of diff.	Below threshold?	Summary	Adjusted P Value
TLK5 vs. HLT2	21765	-24250 to 67780	No	ns	0.5445
TLK5 vs. MAX8	43124	-2891 to 89140	No	ns	0.0703
TLK5 vs. TLE5	78306	32291 to 124321	Yes	***	0.0009
HLT2 vs. MAX8	21359	-24656 to 67374	No	ns	0.5594
HLT2 vs. TLE5	56541	10525 to 102556	Yes	*	0.0137
MAX8 vs. TLE5	35181	-10834 to 81197	No	ns	0.1690

Myeloperoxidase (MPO) Day 3

Tukey's multiple comparisons test	Mean Diff.	95.00% CI of diff.	Below threshold?	Summary	Adjusted P Value
TLK5 vs. HLT2	14210	-19976 to 48395	No	ns	0.6420
TLK5 vs. MAX8	8060	-26125 to 42246	No	ns	0.9052
TLK5 vs. TLE5	33653	-532.5 to 67839	No	ns	0.0544
HLT2 vs. MAX8	-6149	-40335 to 28037	No	ns	0.9544
HLT2 vs. TLE5	19444	-14742 to 53629	No	ns	0.3921
MAX8 vs. TLE5	25593	-8593 to 59779	No	ns	0.1822

Myeloperoxidase (MPO) Day 7

Tukey's multiple comparisons test	Mean Diff.	95.00% CI of diff.	Below threshold?	Summary	Adjusted P Value
TLK5 vs. HLT2	6673	-48148 to 61495	No	ns	0.9850
TLK5 vs. MAX8	-10231	-65053 to 44590	No	ns	0.9495
TLK5 vs. TLE5	50556	-4266 to 105377	No	ns	0.0761
HLT2 vs. MAX8	-16904	-71726 to 37917	No	ns	0.8140
HLT2 vs. TLE5	43883	-10939 to 98704	No	ns	0.1420
MAX8 vs. TLE5	60787	5965 to 115609	Yes	*	0.0272

Myeloperoxidase (MPO) Day 14

Tukey's multiple comparisons test	Mean Diff.	95.00% CI of diff.	Below threshold?	Summary	Adjusted P Value
TLK5 vs. HLT2	-16442	-83827 to 50942	No	ns	0.8963
TLK5 vs. MAX8	-107498	-174883 to -40114	Yes	**	0.0016
TLK5 vs. TLE5	-98723	-166107 to -31338	Yes	**	0.0035
HLT2 vs. MAX8	-91056	-158441 to -23671	Yes	**	0.0067
HLT2 vs. TLE5	-82280	-149665 to -14896	Yes	*	0.0143
MAX8 vs. TLE5	8776	-58609 to 76160	No	ns	0.9817

CXCL1 (KC) Day 1

Tukey's multiple comparisons test	Mean Diff.	95.00% CI of diff.	Below threshold?	Summary	Adjusted P Value
TLK5 vs. HLT2	-166.3	-661.5 to 328.9	No	ns	0.7731
TLK5 vs. MAX8	-2.679	-497.9 to 492.5	No	ns	>0.9999
TLK5 vs. TLE5	573.8	78.56 to 1069	Yes	*	0.0205
HLT2 vs. MAX8	163.6	-331.6 to 658.8	No	ns	0.7814
HLT2 vs. TLE5	740.1	244.8 to 1235	Yes	**	0.0029
MAX8 vs. TLE5	576.5	81.24 to 1072	Yes	*	0.0199

CXCL1 (KC) Day 3

Tukey's multiple comparisons test	Mean Diff.	95.00% CI of diff.	Below threshold?	Summary	Adjusted P Value
TLK5 vs. HLT2	30.61	-13.99 to 75.21	No	ns	0.2393
TLK5 vs. MAX8	57.40	10.09 to 104.7	Yes	*	0.0153
TLK5 vs. TLE5	60.11	15.51 to 104.7	Yes	**	0.0071
HLT2 vs. MAX8	26.79	-20.51 to 74.10	No	ns	0.3913
HLT2 vs. TLE5	29.51	-15.10 to 74.11	No	ns	0.2666
MAX8 vs. TLE5	2.714	-44.59 to 50.02	No	ns	0.9983

CXCL1 (KC) Day 7

Tukey's multiple comparisons test	Mean Diff.	95.00% CI of diff.	Below threshold?	Summary	Adjusted P Value
TLK5 vs. HLT2	1.810	-43.55 to 47.17	No	ns	0.9994
TLK5 vs. MAX8	-8.260	-53.62 to 37.10	No	ns	0.9528
TLK5 vs. TLE5	-28.70	-74.06 to 16.66	No	ns	0.3045
HLT2 vs. MAX8	-10.07	-55.43 to 35.29	No	ns	0.9191
HLT2 vs. TLE5	-30.51	-75.87 to 14.85	No	ns	0.2571
MAX8 vs. TLE5	-20.44	-65.80 to 24.92	No	ns	0.5823

CXCL1 (KC) Day 14

Tukey's multiple comparisons test	Mean Diff.	95.00% CI of diff.	Below threshold?	Summary	Adjusted P Value
TLK5 vs. HLT2	3.369	-216.5 to 223.2	No	ns	TLK5 vs. HLT2
TLK5 vs. MAX8	-5.353	-225.2 to 214.5	No	ns	TLK5 vs. MAX8
TLK5 vs. TLE5	-326.8	-546.6 to -106.9	Yes	**	TLK5 vs. TLE5
HLT2 vs. MAX8	-8.722	-228.6 to 211.1	No	ns	HLT2 vs. MAX8
HLT2 vs. TLE5	-330.1	-550.0 to -110.3	Yes	**	HLT2 vs. TLE5
MAX8 vs. TLE5	-321.4	-541.2 to -101.6	Yes	**	MAX8 vs. TLE5

CCL3 (MIP-1α) Day 1

Tukey's multiple comparisons test	Mean Diff.	95.00% CI of diff.	Below threshold?	Summary	Adjusted P Value
TLK5 vs. HLT2	217.4	57.22 to 377.6	Yes	**	0.0065
TLK5 vs. MAX8	85.00	-75.18 to 245.2	No	ns	0.4502
TLK5 vs. TLE5	277.7	117.5 to 437.8	Yes	***	0.0007
HLT2 vs. MAX8	-132.4	-292.6 to 27.78	No	ns	0.1247
HLT2 vs. TLE5	60.26	-99.92 to 220.4	No	ns	0.7083
MAX8 vs. TLE5	192.7	32.48 to 352.8	Yes	*	0.0159

CCL3 (MIP-1α) Day 3

Tukey's multiple comparisons test	Mean Diff.	95.00% CI of diff.	Below threshold?	Summary	Adjusted P Value
TLK5 vs. HLT2	331.0	-80.64 to 742.6	No	ns	0.1395
TLK5 vs. MAX8	628.2	216.6 to 1040	Yes	**	0.0024
TLK5 vs. TLE5	836.1	424.5 to 1248	Yes	***	0.0001
HLT2 vs. MAX8	297.3	-114.3 to 708.9	No	ns	0.2060
HLT2 vs. TLE5	505.2	93.55 to 916.8	Yes	*	0.0138
MAX8 vs. TLE5	207.9	-203.7 to 619.5	No	ns	0.4913

CCL3 (MIP-1α) Day 7

Tukey's multiple comparisons test	Mean Diff.	95.00% CI of diff.	Below threshold?	Summary	Adjusted P Value
TLK5 vs. HLT2	311.5	-64.05 to 687.0	No	ns	0.1229
TLK5 vs. MAX8	678.5	303.0 to 1054	Yes	***	0.0005
TLK5 vs. TLE5	1431	1056 to 1807	Yes	****	<0.0001
HLT2 vs. MAX8	367.0	-8.519 to 742.6	No	ns	0.0566
HLT2 vs. TLE5	1120	744.2 to 1495	Yes	****	<0.0001
MAX8 vs. TLE5	752.7	377.2 to 1128	Yes	***	0.0002

CCL3 (MIP-1α) Day 14

Tukey's multiple comparisons test	Mean Diff.	95.00% CI of diff.	Below threshold?	Summary	Adjusted P Value
TLK5 vs. HLT2	103.9	-96.86 to 304.6	No	ns	TLK5 vs. HLT2
TLK5 vs. MAX8	-153.3	-354.0 to 47.46	No	ns	TLK5 vs. MAX8
TLK5 vs. TLE5	-134.6	-335.3 to 66.14	No	ns	TLK5 vs. TLE5
HLT2 vs. MAX8	-257.1	-457.8 to -56.40	Yes	*	HLT2 vs. MAX8
HLT2 vs. TLE5	-238.4	-439.1 to -37.71	Yes	*	HLT2 vs. TLE5
MAX8 vs. TLE5	18.69	-182.0 to 219.4	No	ns	MAX8 vs. TLE5

CCL2 (MCP-1) Day 1

Tukey's multiple comparisons test	Mean Diff.	95.00% CI of diff.	Below threshold?	Summary	Adjusted P Value
TLK5 vs. HLT2	41.59	-73.06 to 156.2	No	ns	0.7304
TLK5 vs. MAX8	72.06	-42.59 to 186.7	No	ns	0.3099
TLK5 vs. TLE5	203.3	88.65 to 317.9	Yes	***	0.0006
HLT2 vs. MAX8	30.47	-84.17 to 145.1	No	ns	0.8709
HLT2 vs. TLE5	161.7	47.06 to 276.3	Yes	**	0.0048
MAX8 vs. TLE5	131.2	16.59 to 245.9	Yes	*	0.0222

CCL2 (MCP-1) Day 3

Tukey's multiple comparisons test	Mean Diff.	95.00% CI of diff.	Below threshold?	Summary	Adjusted P Value
TLK5 vs. HLT2	2.286	-54.44 to 59.01	No	ns	0.9994
TLK5 vs. MAX8	-8.003	-64.72 to 48.72	No	ns	0.9770
TLK5 vs. TLE5	-233.4	-290.2 to -176.7	Yes	****	<0.0001
HLT2 vs. MAX8	-10.29	-67.01 to 46.43	No	ns	0.9533
HLT2 vs. TLE5	-235.7	-292.4 to -179.0	Yes	****	<0.0001
MAX8 vs. TLE5	-225.4	-282.2 to -168.7	Yes	****	<0.0001

CCL2 (MCP-1) Day 7

Tukey's multiple comparisons test	Mean Diff.	95.00% CI of diff.	Below threshold?	Summary	Adjusted P Value
TLK5 vs. HLT2	2.078	-54.64 to 58.80	No	ns	0.9996
TLK5 vs. MAX8	-44.17	-100.9 to 12.55	No	ns	0.1578
TLK5 vs. TLE5	-280.1	-336.8 to -223.4	Yes	****	<0.0001
HLT2 vs. MAX8	-46.25	-103.0 to 10.47	No	ns	0.1319
HLT2 vs. TLE5	-282.1	-338.9 to -225.4	Yes	****	<0.0001
MAX8 vs. TLE5	-235.9	-292.6 to -179.2	Yes	****	<0.0001

CCL2 (MCP-1) Day 14

Tukey's multiple comparisons test	Mean Diff.	95.00% CI of diff.	Below threshold?	Summary	Adjusted P Value
TLK5 vs. HLT2	25.30	-42.87 to 93.47	No	ns	0.7168
TLK5 vs. MAX8	-74.13	-142.3 to -5.956	Yes	*	0.0308
TLK5 vs. TLE5	-112.4	-180.6 to -44.26	Yes	**	0.0012
HLT2 vs. MAX8	-99.42	-167.6 to -31.25	Yes	**	0.0036
HLT2 vs. TLE5	-137.7	-205.9 to -69.55	Yes	***	0.0001
MAX8 vs. TLE5	-38.30	-106.5 to 29.87	No	ns	0.4023

IL-6 Day 1

Tukey's multiple comparisons test	Mean Diff.	95.00% CI of diff.	Below threshold?	Summary	Adjusted P Value
TLK5 vs. HLT2	410.7	-443.0 to 1264	No	ns	0.5309
TLK5 vs. MAX8	991.3	137.6 to 1845	Yes	*	0.0202
TLK5 vs. TLE5	2510	1656 to 3364	Yes	****	<0.0001
HLT2 vs. MAX8	580.6	-273.1 to 1434	No	ns	0.2489
HLT2 vs. TLE5	2099	1246 to 2953	Yes	****	<0.0001
MAX8 vs. TLE5	1519	664.9 to 2372	Yes	***	0.0006

IL-6 Day 3

Tukey's multiple comparisons test	Mean Diff.	95.00% CI of diff.	Below threshold?	Summary	Adjusted P Value
TLK5 vs. HLT2	154.5	78.24 to 230.7	Yes	***	0.0001
TLK5 vs. MAX8	185.0	108.8 to 261.2	Yes	****	<0.0001
TLK5 vs. TLE5	189.2	113.0 to 265.4	Yes	****	<0.0001
HLT2 vs. MAX8	30.51	-45.70 to 106.7	No	ns	0.6680
HLT2 vs. TLE5	34.73	-41.48 to 110.9	No	ns	0.5738
MAX8 vs. TLE5	4.219	-71.99 to 80.43	No	ns	0.9985

IL-6 Day 7

Tukey's multiple comparisons test	Mean Diff.	95.00% CI of diff.	Below threshold?	Summary	Adjusted P Value
TLK5 vs. HLT2	9.543	-17.15 to 36.24	No	ns	0.7389
TLK5 vs. MAX8	-0.1570	-26.85 to 26.54	No	ns	>0.9999
TLK5 vs. TLE5	8.797	-17.90 to 35.49	No	ns	0.7826
HLT2 vs. MAX8	-9.700	-36.40 to 17.00	No	ns	0.7294
HLT2 vs. TLE5	-0.7457	-27.44 to 25.95	No	ns	0.9998
MAX8 vs. TLE5	8.954	-17.74 to 35.65	No	ns	0.7736

IL-6 Day 14

Tukey's multiple comparisons test	Mean Diff.	95.00% CI of diff.	Below threshold?	Summary	Adjusted P Value
TLK5 vs. HLT2	-0.08951	-3.882 to 3.703	No	ns	0.9999
TLK5 vs. MAX8	-0.1118	-3.396 to 3.172	No	ns	0.9996
TLK5 vs. TLE5	-3.873	-7.157 to -0.5887	Yes	*	0.0189
HLT2 vs. MAX8	-0.02232	-3.815 to 3.770	No	ns	>0.9999
HLT2 vs. TLE5	-3.783	-7.576 to 0.008919	No	ns	0.0506
MAX8 vs. TLE5	-3.761	-7.045 to -0.4768	Yes	*	0.0228

TNF-α Day 1

Tukey's multiple comparisons test	Mean Diff.	95.00% CI of diff.	Below threshold?	Summary	Adjusted P Value
TLK5 vs. HLT2	18.04	-21.13 to 57.21	No	ns	0.5656
TLK5 vs. MAX8	54.26	15.09 to 93.43	Yes	**	0.0055
TLK5 vs. TLE5	76.48	37.31 to 115.7	Yes	***	0.0002
HLT2 vs. MAX8	36.22	-2.952 to 75.39	No	ns	0.0751
HLT2 vs. TLE5	58.44	19.27 to 97.61	Yes	**	0.0030
MAX8 vs. TLE5	22.22	-16.95 to 61.39	No	ns	0.3942

TNF-α Day 3

Tukey's multiple comparisons test	Mean Diff.	95.00% CI of diff.	Below threshold?	Summary	Adjusted P Value
TLK5 vs. HLT2	46.45	4.409 to 88.49	Yes	*	0.0279
TLK5 vs. MAX8	62.38	20.34 to 104.4	Yes	**	0.0031
TLK5 vs. TLE5	174.4	132.4 to 216.4	Yes	****	<0.0001
HLT2 vs. MAX8	15.93	-26.11 to 57.97	No	ns	0.7039
HLT2 vs. TLE5	128.0	85.92 to 170.0	Yes	****	<0.0001
MAX8 vs. TLE5	112.0	69.99 to 154.1	Yes	****	<0.0001

TNF-α Day 7

Tukey's multiple comparisons test	Mean Diff.	95.00% CI of diff.	Below threshold?	Summary	Adjusted P Value
TLK5 vs. HLT2	6.283	-29.61 to 42.17	No	ns	0.9577
TLK5 vs. MAX8	19.49	-16.40 to 55.37	No	ns	0.4310
TLK5 vs. TLE5	106.5	70.58 to 142.4	Yes	****	<0.0001
HLT2 vs. MAX8	13.20	-22.69 to 49.09	No	ns	0.7220
HLT2 vs. TLE5	100.2	64.29 to 136.1	Yes	****	<0.0001
MAX8 vs. TLE5	86.98	51.09 to 122.9	Yes	****	<0.0001

TNF-α Day 14

Tukey's multiple comparisons test	Mean Diff.	95.00% CI of diff.	Below threshold?	Summary	Adjusted P Value
TLK5 vs. HLT2	2.125	-5.840 to 10.09	No	ns	0.8697
TLK5 vs. MAX8	-9.195	-17.16 to -1.230	Yes	*	0.0210
TLK5 vs. TLE5	-6.393	-14.36 to 1.572	No	ns	0.1405
HLT2 vs. MAX8	-11.32	-19.29 to -3.355	Yes	**	0.0045
HLT2 vs. TLE5	-8.518	-16.48 to -0.5527	Yes	*	0.0340
MAX8 vs. TLE5	2.802	-5.163 to 10.77	No	ns	0.7480

IL-1β Day 1

Tukey's multiple comparisons test	Mean Diff.	95.00% CI of diff.	Below threshold?	Summary	Adjusted P Value
TLK5 vs. HLT2	-6.199	-25.23 to 12.83	No	ns	0.7885
TLK5 vs. MAX8	-0.7954	-19.83 to 18.24	No	ns	0.9994
TLK5 vs. TLE5	6.683	-12.35 to 25.72	No	ns	0.7491
HLT2 vs. MAX8	5.403	-13.63 to 24.44	No	ns	0.8478
HLT2 vs. TLE5	12.88	-6.152 to 31.91	No	ns	0.2526
MAX8 vs. TLE5	7.478	-11.56 to 26.51	No	ns	0.6805

IL-1β Day 3

Tukey's multiple comparisons test	Mean Diff.	95.00% CI of diff.	Below threshold?	Summary	Adjusted P Value
TLK5 vs. HLT2	20.17	-0.1632 to 40.51	No	ns	0.0522
TLK5 vs. MAX8	25.28	4.948 to 45.62	Yes	*	0.0126
TLK5 vs. TLE5	48.79	28.46 to 69.13	Yes	****	<0.0001
HLT2 vs. MAX8	5.112	-15.22 to 25.45	No	ns	0.8880
HLT2 vs. TLE5	28.62	8.285 to 48.95	Yes	**	0.0049
MAX8 vs. TLE5	23.51	3.173 to 43.84	Yes	*	0.0208

IL-1β Day 7

Tukey's multiple comparisons test	Mean Diff.	95.00% CI of diff.	Below threshold?	Summary	Adjusted P Value
TLK5 vs. HLT2	10.67	0.6229 to 20.71	Yes	*	0.0355
TLK5 vs. MAX8	11.08	1.033 to 21.12	Yes	*	0.0282
TLK5 vs. TLE5	31.60	21.55 to 41.64	Yes	****	<0.0001
HLT2 vs. MAX8	0.4104	-9.633 to 10.45	No	ns	0.9994
HLT2 vs. TLE5	20.93	10.89 to 30.97	Yes	***	0.0001
MAX8 vs. TLE5	20.52	10.48 to 30.56	Yes	***	0.0001

IL-1β Day 14

Tukey's multiple comparisons test	Mean Diff.	95.00% CI of diff.	Below threshold?	Summary	Adjusted P Value
TLK5 vs. HLT2	-0.5344	-2.685 to 1.616	No	ns	0.8891
TLK5 vs. MAX8	-0.6339	-2.662 to 1.394	No	ns	0.8044
TLK5 vs. TLE5	-2.422	-4.450 to -0.3947	Yes	*	0.0170
HLT2 vs. MAX8	-0.09952	-2.250 to 2.051	No	ns	0.9991
HLT2 vs. TLE5	-1.888	-4.038 to 0.2628	No	ns	0.0953
MAX8 vs. TLE5	-1.788	-3.816 to 0.2393	No	ns	0.0933

G-CSF Day 1

Tukey's multiple comparisons test	Mean Diff.	95.00% CI of diff.	Below threshold?	Summary	Adjusted P Value
TLK5 vs. HLT2	-1755	-2764 to -746.8	Yes	***	0.0007
TLK5 vs. MAX8	1722	713.2 to 2730	Yes	***	0.0009
TLK5 vs. TLE5	2963	1954 to 3971	Yes	****	<0.0001
HLT2 vs. MAX8	3477	2469 to 4486	Yes	****	<0.0001
HLT2 vs. TLE5	4718	3709 to 5727	Yes	****	<0.0001

MAX8 vs. TLE5	1241	232.1 to 2249	Yes	*	0.0136

G-CSF Day 3

Tukey's multiple comparisons test	Mean Diff.	95.00% CI of diff.	Below threshold?	Summary	Adjusted P Value
TLK5 vs. HLT2	283.6	-314.9 to 882.1	No	ns	0.5432
TLK5 vs. MAX8	512.8	-85.66 to 1111	No	ns	0.1070
TLK5 vs. TLE5	797.8	199.3 to 1396	Yes	**	0.0075
HLT2 vs. MAX8	229.3	-369.2 to 827.8	No	ns	0.6969
HLT2 vs. TLE5	514.2	-84.26 to 1113	No	ns	0.1057
MAX8 vs. TLE5	285.0	-313.5 to 883.5	No	ns	0.5393

G-CSF Day 7

Tukey's multiple comparisons test	Mean Diff.	95.00% CI of diff.	Below threshold?	Summary	Adjusted P Value
TLK5 vs. HLT2	-23.87	-123.3 to 75.52	No	ns	0.8985
TLK5 vs. MAX8	-23.14	-122.5 to 76.26	No	ns	0.9064
TLK5 vs. TLE5	29.04	-70.35 to 128.4	No	ns	0.8337
HLT2 vs. MAX8	0.7327	-92.98 to 94.45	No	ns	>0.9999
HLT2 vs. TLE5	52.92	-40.79 to 146.6	No	ns	0.3937
MAX8 vs. TLE5	52.19	-41.53 to 145.9	No	ns	0.4053

G-CSF Day 14

Tukey's multiple comparisons test	Mean Diff.	95.00% CI of diff.	Below threshold?	Summary	Adjusted P Value
TLK5 vs. HLT2	-4.705	-690.7 to 681.3	No	ns	>0.9999
TLK5 vs. MAX8	1.566	-684.5 to 687.6	No	ns	>0.9999
TLK5 vs. TLE5	-956.1	-1642 to -270.0	Yes	**	0.0053
HLT2 vs. MAX8	6.271	-679.7 to 692.3	No	ns	>0.9999
HLT2 vs. TLE5	-951.4	-1637 to -265.3	Yes	**	0.0055
MAX8 vs. TLE5	-957.6	-1644 to -271.6	Yes	**	0.0052

CCL5 RANTES Day 1

Tukey's multiple comparisons test	Mean Diff.	95.00% CI of diff.	Below threshold?	Summary	Adjusted P Value
TLK5 vs. HLT2	0.5697	-1.973 to 3.112	No	ns	0.9171
TLK5 vs. MAX8	-4.562	-7.104 to -2.019	Yes	***	0.0005
TLK5 vs. TLE5	-1.219	-3.762 to 1.323	No	ns	0.5335
HLT2 vs. MAX8	-5.131	-7.674 to -2.589	Yes	***	0.0002
HLT2 vs. TLE5	-1.789	-4.332 to 0.7536	No	ns	0.2242
MAX8 vs. TLE5	3.342	0.7994 to 5.885	Yes	**	0.0083

CCL5 RANTES Day 3

Tukey's multiple comparisons test	Mean Diff.	95.00% CI of diff.	Below threshold?	Summary	Adjusted P Value
TLK5 vs. HLT2	0.01392	-0.5559 to 0.5837	No	ns	0.9999
TLK5 vs. MAX8	-0.5455	-1.115 to 0.02428	No	ns	0.0630
TLK5 vs. TLE5	-0.2948	-0.8646 to 0.2750	No	ns	0.4713
HLT2 vs. MAX8	-0.5594	-1.129 to 0.01035	No	ns	0.0552
HLT2 vs. TLE5	-0.3087	-0.8785 to 0.2611	No	ns	0.4327
MAX8 vs. TLE5	0.2507	-0.3191 to 0.8205	No	ns	0.6005

CCL5 RANTES Day 7

Tukey's multiple comparisons test	Mean Diff.	95.00% CI of diff.	Below threshold?	Summary	Adjusted P Value
TLK5 vs. HLT2	0.09916	-1.737 to 1.935	No	ns	0.9986
TLK5 vs. MAX8	-3.497	-5.333 to -1.662	Yes	***	0.0003
TLK5 vs. TLE5	0.2869	-1.549 to 2.123	No	ns	0.9692
HLT2 vs. MAX8	-3.596	-5.432 to -1.761	Yes	***	0.0002
HLT2 vs. TLE5	0.1878	-1.648 to 2.023	No	ns	0.9909
MAX8 vs. TLE5	3.784	1.948 to 5.620	Yes	***	0.0001

CCL5 RANTES Day 14

Tukey's multiple comparisons test	Mean Diff.	95.00% CI of diff.	Below threshold?	Summary	Adjusted P Value
TLK5 vs. HLT2	-7.483	-15.36 to 0.3952	No	ns	0.0657
TLK5 vs. MAX8	-10.56	-18.44 to -2.682	Yes	**	0.0072
TLK5 vs. TLE5	4.121	-3.757 to 12.00	No	ns	0.4622
HLT2 vs. MAX8	-3.077	-10.95 to 4.801	No	ns	0.6844
HLT2 vs. TLE5	11.60	3.726 to 19.48	Yes	**	0.0033
MAX8 vs. TLE5	14.68	6.803 to 22.56	Yes	***	0.0004
